# Heteroaromatic Hybrid Benzimidazole/Oxadiazole (BZ/OZ) Ligand and Its Sm(III) Complex: Study of Their Antibacterial Activity, Toxicological Prediction and Interaction with Different Model Membranes

**DOI:** 10.3390/biom15111568

**Published:** 2025-11-07

**Authors:** Alberto Aragón-Muriel, Alessio Ausili, Luciana Sampaio Lima, Cleydson B. R. Santos, David Morales-Morales, Dorian Polo-Cerón

**Affiliations:** 1 Grupo de Investigaciones Bioquímicas (GIB), Departamento de Química, Universidad del Magdalena, Santa Marta 470004, Colombia; aaragonm@unimagdalena.edu.co; 2 Departamento de Bioquímica y Biología Molecular (A), Facultad de Veterinaria, International Campus of Excellence Mare Nostrum, Universidad de Murcia, Apartado. 4021, E-30100 Murcia, Spain; 3 Laboratório de Modelagem e Química Computacional, Universidade Federal do Amapá, Macapá 68903-419, Amapá, Brazil; lucianasampaio@unifap.br (L.S.L.); breno@unifap.br (C.B.R.S.); 4 Instituto de Química, Universidad Nacional Autónoma de México, Circuito Exterior S/N, Ciudad Universitaria, Alcaldía Coyoacán, Ciudad de México C.P. 04510, Mexico; damor@unam.mx; 5 Laboratorio de Investigación en Catálisis y Procesos (LICAP-Bioinorgánica), Departamento de Química, Facultad de Ciencias Naturales y Exactas, Universidad del Valle, Calle 13 No. 100-00, Santiago de Cali 76001, Colombia

**Keywords:** heteroaromatic hybrid, antibacterial activity, model membranes, biophysical study, toxicological prediction, coordination complex

## Abstract

Two heteroaromatic hybrid compounds were synthesized and characterized using various analytical techniques. The results indicate that the benzimidazole/oxadiazole (BZ/OZ) metal derivative exhibits a tridentate coordination mode, where the carbonyl, imidazole and oxadiazole groups participate in coordination with the metal, in a ratio of 2:1 of the ligand to the metal. The antibacterial activities of the organic ligand and its metal complex were determined by in vitro tests against both Gram-positive bacterial strains and Gram-negative bacterial strains using the broth microdilution method. The metal complex showed greater antibacterial activities compared to the precursor ligand against all evaluated microorganisms. The results obtained through in silico predictions revealed significant toxicological differences among the analyzed molecules, suggesting special attention in the use of the ligand due to its possible carcinogenicity in mice and a need for structural modifications in the complex to reduce its carcinogenicity and toxicity. Furthermore, a biophysical study of the interaction of the BZ/OZ derivatives with different model membranes was explored through differential scanning calorimetry (DSC), simultaneous small- and wide-angle X-ray diffraction (SAXD and WAXD) and infrared spectroscopy (FT-IR). The results indicate that the compounds influenced membrane properties without significantly altering the lamellar organization. The findings suggest potential applications in understanding lipid interactions, elucidating toxicology and developing antibacterial agents.

## 1. Introduction

The increasing bacterial resistance to multiple drugs represents a significant challenge for public health, thereby requiring urgent research and development of innovative strategies to effectively combat multidrug-resistant microbial agents that cause multiple infectious diseases [1,2,3]. Molecular hybridization is a strategy that is a noteworthy approach in medicinal chemistry for the development of novel antimicrobials. This approach involves the combination of various pharmacophores into a single molecule [4,5]. Hybrid molecules exhibiting potential biological activity have emerged as a highly interesting alternative, since they integrate the characteristics of different bioactive compounds with multiple mechanisms of biological action, which could enhance therapeutic efficacy and reduce the adverse effects associated with first-line antimicrobial drugs [6,7]; among them are reported organic molecules derived from chloroquinolines, fluoroquinolones, benzothiazoles, benzimidazoles, benzoxazoles, pyrimidines and pharmaceutical scaffolds [7,8,9,10]. However, inorganic materials have also been explored as alternatives, including metallic nanoparticles [6,11] and coordination complexes with ligands such as phosphazenes, curcumin, quinolines and sulfonamides [12,13,14,15]. These studies demonstrate that, for example, the Co(II) ion presents better antibacterial activity with a ligand derived from a natural product compared to its Cu(II) analog compound [13], while for the Quinoline–Sulfonamide ligand the activity of the Cu(II) complex was better compared to other ions including Co(II) [14]; the differences are explained in relation to the geometry, charge transfer and redox properties that condition the effect. While in the case of ligands containing phosphazenes, there is the advantage of obtaining multidentate and highly functionalizable scaffolds that facilitate the design of derivatives with strong interactions with metals and with multiple modes of action [12].

Heteroaromatic frameworks of benzimidazole (BZ) and oxadiazole (OZ), have been recognized for their multiple chemical structures and different pharmacological properties [16], which include antiviral [17], antihelminthic [18], anti-inflammatory [19], antiproliferative [20], anticancer [21,22] and antimicrobial [23,24]. Thus, the combined BZ/OZ heterocyclic derivatives are of great interest in medicinal chemistry due to their ability to coordinate with metal centers, resulting in the formation of an almost limitless number of complexes with a plethora of structures and conformations. This enhanced antimicrobial property being the result of their molecular architectures [25]. Furthermore, challenges persist in the synthesis of BZ/OZ hybrid derivatives, including limitations related to solubility, structural stability and incomplete characterization of rare earth complexes, which highlights the novelty of this work.

The molecular mechanisms that underline the diverse pharmacological properties and biological actions of BZ/OZ-derived compounds frequently involve the inhibition of essential enzymes, interference with DNA replication, disruption of mitochondrial function and cellular signaling pathways [20,26,27,28,29,30,31,32]. In particular, BZ/OZ derivatives have demonstrated cytotoxic effects against fungi and Gram-positive and Gram-negative bacteria [33,34,35,36], although their mechanisms of action remain unclear. The heterocyclic derivatives interact with the phospholipids present in the lipid bilayer of the bacterial membrane, resulting in destabilization and cell disintegration as the most frequently mechanism described in the literature [37,38,39,40].

Within the studies of antibacterial activity of lanthanide complexes, Sm(III) compounds have been underexplored; however, there are reports that demonstrate that these complexes can have greater antimicrobial activity than free ligands and show affinity for biomolecules (DNA/BSA) [41,42]. Some plausible mechanisms include interaction with bacterial membranes by electrostatic interactions and displacement/labilization of cellular ions, and generation of reactive species (ROS) that support bactericidal or bacteriostatic activity [43]. It has been reported that the coordination of ligands with donor atoms (N or S) to samarium metal centers can (i) increase the apparent lipophilicity of the system, facilitating membrane interaction; (ii) rigidify conformations that favor packing against the bilayer; and (iii) create localized cationic centers that interact electrostatically with bacterial anionic surfaces (LPS, teichoic acids) [44,45].

**Figure 1:**
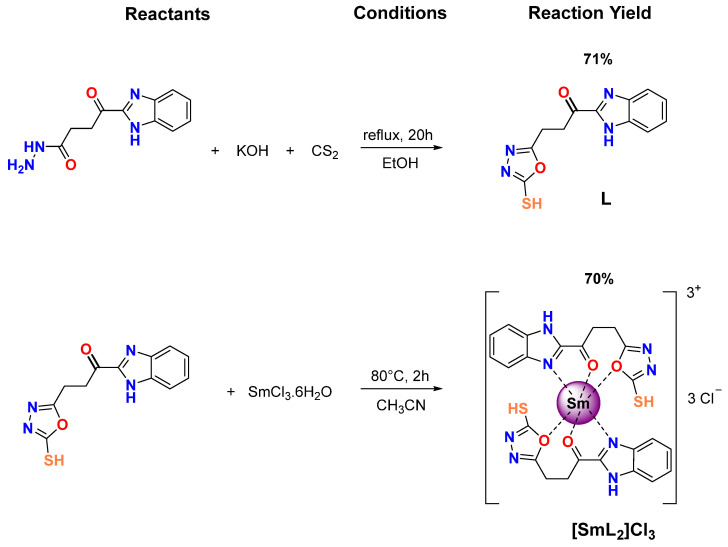
Synthesis of BZ/OZ derivatives: organic ligand (L) and its samarium complex ([SmL_2_]Cl_3_).

Thus, in this work, we present the synthesis and characterization of a novel hybrid derivative of heteroaromatic BZ/OZ ligand and its Sm(III) complex (Figure 1), as part of our ongoing attempt to develop novel compounds with biological activity [46,47,48,49]. To evaluate their potential antimicrobial activity, tests were performed against Gram-positive and Gram-negative bacterial strains. In addition, a detailed biophysical study was conducted using techniques, such as differential scanning calorimetry (DSC), simultaneous small- and wide-angle X-ray diffraction (SAXD and WAXD) and infrared spectroscopy (FT-IR), to explore the effects of the interaction of both the BZ/OZ derivative and its complex on the thermotropic and structural properties of lipid membrane models, such as 1,2-dimyristoyl-sn-glycero-3-phosphocholine (DMPC), 1,2-dimyristoyl-sn-glycero-3-phospho-rac-(1-glycerol) (DMPG) and 1-palmitoyl-2-oleoyl-sn-glycero-3-phosphoethanolamine (POPE). The results obtained provide valuable information on the influence of these compounds on the stability and properties of membranes, which could offer new approaches for the design of more effective antimicrobial therapies.

## 2. Materials and Methods

With the exception of 4-(1H-benzo[d]imidazol-2-yl)-4-oxobutanehydrazide, all reagents and solvents used for the synthesis of the BZ/OZ derivatives were purchased from Sigma–Aldrich (St. Louis, MO, USA) and were used directly without prior purification. The phospholipids used in this study were 1,2-dimyristoyl-sn-glycero-3-phosphocholine (DMPC), 1,2-dimyristoyl-sn-glycero-3-phospho-rac-(1-glycerol) (DMPG) and 1-palmitoyl-2-oleoyl-sn-glycero-3-phosphoethanolamine (POPE). These products were purchased from Avanti Polar Lipids (Alabaster, AL, USA). The ^1^H and ^13^C NMR spectra were obtained with a 400 MHz Bruker Ultra Shield spectrometer, using DMSO-*d*_6_ as deuterated solvent. The following abbreviations were used: s = singlet; d = doublet; t = triplet; and m = multiplet. The infrared spectra of BZ/OZ derivatives were taken on the SHIMADZU IR Affinity-1 spectrophotometer (Shimadzu, Miyazaki, Japan) with KBr pellet and Thermo scientific (Waltham, MA, USA) Nicolet TM 6700 (ATR). The study of the percentage composition of the products was performed, with an accepted tolerance of ±2% on carbon (C), hydrogen (H), nitrogen (N) and sulfur (S), using the Thermo Flash EA 1112 Series elemental analyzer (Waltham, MA, USA), and the oxygen (O) content was obtained by subtraction. The percentage of metal was determined by complexometric titration with EDTA. The mass spectra of L was performed on the SHIMADZU-GCMS-QP2010 mass spectrometer (Shimadzu, Miyazaki, Japan) using electron impact ionization (EI) at 70 eV. The melting points of the compounds were determined on a Stuart^®^ SMP10 melting point meter (St. Louis, MO, USA). The conductivity of the metal complex was determined in ethanol (1 × 10^−3^ M) using an OrionTM 131S (Thermo Fischer Scientific, Waltham, MA, USA).

### 2.1. Synthesis of BZ/OZ Derivatives, L and [SmL_2_]Cl_3_

The new BZ/OZ ligand (L) was made using the method described by Husain [50] (Figure 1). A total of 0.23 g/1.00 mmol of 4-(1H-benzo[*d*]imidazol-2-yl)-4-oxobutanehydrazide (obtained previously based on the literature [50,51]) was dissolved in 10 mL of ethanol 96%, and a solution of KOH ≥ 85% (500 µL, 0.1 mol∙L^−1^, 1.00 mmol) was slowly added dropwise with stirring over 30 min at 50 °C. Carbon disulphide ≥ 99% (0.82 0.1 mol∙L^−1^, 8.20 mmol) was added, and the mixture was refluxed for 20 additional hours. The resulting solid was acidified (pH = 2). The solid formed was filtered and washed with a mixture of methanol–water (1:2) to provide the BZ/OZ derivative as a yellow solid, which did not require further purification. Yield: (190 mg, 71%). m.p.: 253–255 °C; Anal. calcd. For C_12_H_10_N_4_O_2_S: C, 52.5; N, 20.4; H, 3.7; and S, 11.7. Found: C, 51.8; N, 20.8; H, 3.7; and S, 12.1. IR (ATR, cm^−1^). υ: 2830 (S-H), 1640 (C=O), 1580 (C=N), 1250 and 1120 (C-O-C). ^1^H-NMR (DMSO-*d*_6_, 400 MHz): δ_H_ 3.21 (s, 4H, H-11 y H-12), 7.32 (m, *J* = 8.0 2H, H-5 y H-6), 7.51 (t, *J* = 8.0, 1H, H-4), 7.71 (d, *J* = 8.0, 1H, H-3), 12.42 (s, 1H, H-1) and 14.28 (s, 1H, H-18). ^13^C-NMR (DMSO-*d*_6_, 100 MHz): δ C21.8 (C12), 28.62 (C11), 115.89 (C5), 123.65 (C6), 128.56 (C3), 130.18 (C4), 131.83 (C2), 132.29 (C7), 154.91 (C13), 159.5 (C9), 164.26 (C10) and 178.10 (C16). MS electron impact (*m*/*z*, 70 eV): 274 (M+).

A solution of Samarium(III)-chloride hexahydrate ≥ 99.99% (SmCl_3_·6H_2_O, 182.4 mg, 0.5 mmol) in acetonitrile ≥ 99.5% (5.0 mL) was added dropwise into 10 mL of acetonitrile solution of L (232 mg, 1.00 mmol), and the formation of a precipitate was observed. The mixture was kept under constant stirring for 2 h at 80 °C. Finally, the brown solid was isolated by filtration, washed with a mixture of methanol–water (1:1), and then dried in a desiccator for 3 days (Figure 1). Yield: (310 mg, 70%). m.p.: 265–267 °C; Anal. calcd. For C_24_H_20_N_8_O_4_S_2_SmCl_3_: C, 35.8; N, 13.9; H, 2.5; S, 7.9; and Sm, 18.7. Found: C, 34.9; N, 13.0; H, 3.1; S, 7.5; and Sm, 17.9. IR (ATR, cm^−1^), 2755 (S-H), 1665 (C=O), 1536 (C=N), 1269 and 1200 (C-O-C). Λ (DMSO, 28 °C) (Ω^−1^∙cm^2^∙mol^−1^): 204.

### 2.2. Antibacterial Activity: Determination of the Minimum Inhibitory Concentration

The minimum inhibitory concentration (MIC) was determined using the microdilution method [52] on five Gram-negative bacterial strains (*E. coli* ATCC 25922, *K. pneumoniae* ATCC 2146 and clinical strains *S. dysenteriae* and *Salmonella*) and two Gram-positive bacterial strains (*B. subtilis* ATCC 6633 and *S. aureus* ATCC 6538). The bacterial strains were replicated on a chocolate agar culture under aerobic conditions at 37 °C for 24 h. After preparing the inoculum and isolating the bacterial colonies, they were suspended in Mueller–Hinton broth until obtaining a solution with a turbidity equivalent to 0.5 McFarland. The stock solution of the bacteria was then prepared at an approximate concentration of 1 × 10^8^ colony-forming units (CFU) per milliliter. Subsequently, an aliquot of the stock solution was taken and dissolved in Mueller–Hinton broth, thereby obtaining the working solution for each pathogenic microorganism. The study compounds were plated in triplicate in 96-well plates. The positive control (Mueller–Hinton broth) was deposited in column 1, the negative control (Mueller–Hinton broth and bacterial solution) in column 12, and the BZ/OZ derivatives were deposited in the remaining wells in a concentration range between 1.95 µg mL^−1^ and 1000 µg mL^−1^. Finally, each of the plates was incubated at 37 °C for a period of 24 h under aerobic conditions to obtain the MIC (µg·mL^−1^) results. Ciprofloxacin > 98% (CP) and silver nitrate ≥ 99.0% (AgNO_3_) were used as control antibiotics.

### 2.3. Model Membranes Preparation

For all experiments and techniques, aqueous dispersions of phospholipids were used as model membranes. The phospholipids used to generate these samples were DMPC ≥ 98.0%, DMPG ≥ 98.0% and POPE ≥ 95% and were chosen to mimic mammalian and bacterial cell membranes. More specifically, DMPC is a zwitterionic phospholipid and was used to generate a membrane with a neutral, simple and stable surface charge to simulate the eukaryotic plasma membrane [53,54]. On the other hand, DMPG, which is an anionic phospholipid, was chosen as a study model to mimic the negative charge present in the outer membrane leaflet typical of Gram-positive bacteria and also to a lesser extent in the internal plasma membrane of Gram-negative bacteria [55]. Finally, POPE was employed because, together with phosphatidylglycerol, phosphatidylethanolamine is the most abundant phospholipid in bacterial membranes [55]. For the latter phospholipid, POPE was preferred to DMPE, for practical reasons, as the phase transition temperature of POPE is within our experimental temperature parameters.

Phospholipid dispersions with and without BZ/OZ derivatives (L and [SmL_2_]Cl_3_), were prepared by hydrating the required amounts of DMPC, DMPG or POPE and the compounds in a 20 mM Hepes, 100 mM KCl buffer, pH 7.5. The desired amounts of phospholipids (depending on the type of experiment) were mixed from chloroform/methanol solutions (2:1) and the compound BZ/OZ derivatives from ethanol solutions. The solvents were carefully evaporated under a flow of nitrogen, and the residual traces of solvent were removed by high vacuum drying for at least 3 h. The buffer was added to the dried samples, and they were vortexed until a homogeneous suspension of phospholipids was obtained, always keeping the temperature above the gel to liquid-crystalline phasetransition temperature of the phospholipids. The concentration of the phospholipids was determined by the Böttcher method [56].

### 2.4. Differential Scanning Calorimetry Studies

DSC samples were prepared as described above using 2.95 µmoles of phospholipids in the absence and presence of L and [SmL_2_]Cl_3_ compounds. The phospholipid/compound molar ratios were 100:1, 50:1 and 20:1 for both L and [SmL_2_]Cl_3_. In the case of L, a further concentration of 10:1 was measured to equate the total concentration of L as two L molecules linked by a samarium atom are present in one [SmL_2_]Cl_3_ molecule. To prepare the lipid dispersions, the samples were hydrated in a 2 mL buffer to obtain a phospholipidic concentration of about 1 mg/mL. Measurements were carried out with a high-resolution MicroCal MC-2 microcalorimeter (Microcal, Northampton, MA, USA), employing in the reference cell the same buffer used to prepare the phospholipidic suspension. Samples and references were degassed for 10 min before they were loaded into the microcalorimeter. The temperature range of the scans was between 10 and 40 °C for the DMPC and DMPG samples and up to 75 °C for POPE, so as to observe the formation of the hexagonal phase and the effect of the compounds on it, while the scan-rate was 1 °C/min for all samples. Three consecutive heating scans were performed for each sample to ensure the scan-to-scan reproducibility, and the last scan was used for data analysis, which was performed with the Microcal Origin 5.0 software. A buffer vs. buffer thermogram was subtracted from all sample thermograms before data processing. The parameters calculated from the final thermograms were the transition temperature and the enthalpy. The onset and end temperatures of the transition were the assumed temperature values corresponding to 10% of the maximum peak height. While the transition temperature corresponded to the maximum peak height [57].

### 2.5. FTIR Studies

An amount of 5.9 µmoles of phospholipids was used in FTIR experiments. The samples, prepared as described above, were studied in the absence and presence of BZ/OZ derivatives. In this method, the highest concentrations of the compounds used in the DSC measurements were used to observe their effect on the model membranes. For L the molar ratio was 10:1 (lipid/compound), while for [SmL_2_]Cl_3_ it was 20:1 (lipid/compound). The dried mixtures were hydrated in a total volume of 30 µL buffer and the lipid dispersions were generated. Samples were placed directly into a GS20500 temperature cell equipped with two CaF_2_ windows and a 25 µm spacer mounted in a water heating jacket cell holder GS20710 (Graseby-Specac Ltd., Orpington, Kent, UK). The FTIR spectra were recorded with a Bruker Vector 22 spectrometer (Bruker, Etlingen, Germany) equipped with a liquid nitrogen-cooled MCT detector with a normal triangular apodization function. The software used to collect the spectra was “Opus 6” provided by Bruker. The water vapor inside the device was eliminated by purging it during at least 24 h with a dry air flow. Spectra were collected from 8 to 40 °C with temperature ranges of 2 °C, each recording 128 scans with a nominal resolution of 2 cm^−1^. A spectrum of the buffer was also taken under the same conditions and subtracted from the samples’ spectra. Data processing was performed using OriginPro 8.5 software. The transition temperature was calculated by best-fitting the data with the Boltzmann sigmoidal function and taking the flexion point as transition temperature (Tm).

### 2.6. X-Ray Studies

Simultaneous wide-angle (WAXD) and small-angle (SAXD) X-ray diffraction measurements were performed on a Kratky compact camera (Mbraum-Graz Optical Systems, Graz, Austria) equipped with two coupled linear position detectors (PSD; MBraum, Garching, Germany). A radiation Kα(Cu) produced by a Philips PW 3830 lightning generator (Philips, Eindhoven, The Netherlands) operating at 50 kV and 30 mA was used. The calibration of the detector was carried out using silver stearate (small angle region, d-spacing at 48.8 Å) as reference material and lupolen (wide-angle region, d-spacing at 4.12 Å).

The samples for X-ray diffraction analysis were prepared using a phospholipid amount of 29.5 µmol and a corresponding quantity of L and [SmL_2_]Cl_3_ in order to obtain molar ratios of 10:1 and 20:1, respectively. The lipid suspensions were deposited in a steel sample holder with cellophane windows, which provides a good thermal contact with the Peltier thermostat. The usual exposure times were 5 min, with a waiting time before measurement of 10 min to equilibrate the temperature. The samples were analyzed at three different temperatures: 8, 15 and 30 °C for DMPC and DMPG, and 15, 35 and 70 °C for POPE. SAXS data were also analyzed with the GAP (Global Analysis Program) version 1.3 of G. Pabst [58]. This program can calculate the lamellar repeat distance d, the distance between the phosphate groups of the polar heads between the two leaflets of the lipid bilayer dHH, the width of the membrane dB (considering as an excellent approximation the width of the phospholipidic head groups of 6 Å) and the width of the water layer (expressed as dW = d − dB) from a complete analysis of the range-q of SAXS patterns [59].

### 2.7. In Silico Study

Toxicity prediction tests were performed using Discovery Studio 2015 software (Accelrys, Inc., San Diego, CA, USA), via the Toxicity Prediction function (BIOVIA), by Komputer Assisted Technology (TOPKAT) [60], based on the 2D molecular structure. Quantitative structure–toxicity relationship models with cross-validation were used to evaluate the toxicological properties of carcinogenicity in female mice and rats, Ames mutagenicity, skin irritation, and sensitization. Toxicity risk prediction calculations were performed via TOPKAT and measured the parameters of oral rate LD 50 of body weight, EC 50 (g/kg) in Daphnia magna (mg/L), rat chronic LOAEL (g/kg of body weight), LC 50 in mice (g/L). The carcinogenic potential was predicted using the parameters TD 50 (mg/kg of body weight/mouse-day/rat) and RMTD (maximum tolerated dose in rats-g/kg of body weight).

## 3. Results and Discussion

### 3.1. Synthesis and Characterization of BZ/OZ Derivatives L and [SmL_2_]Cl_3_

The BZ/OZ ligand (L) was prepared in an ethanolic medium with an excess of carbon sulfide, producing the 1,3,4-oxadizole ring by intramolecular attack of the electrons from the oxygen atom to the carbon of the thiocarbonyl intermediate [50]. The ligand was isolated as a yellow solid, stable in air, soluble in DMSO and DMF, partially soluble in ethanol and methanol but insoluble in water. The ligand was further characterized by melting point, elemental analysis, IR spectroscopy, NMR and mass spectrometry. Figure S1 shows the FT-IR spectrum of both the free BZ/OZ ligand and its Sm(III) derivative, with the main bands assigned to the functional groups of these compounds. The benzimidazole ring was evidenced by the presence of the stretching vibration bands υ(N-H) and υ(C=N) at 3100 cm^−1^ and 1580 cm^−1^, respectively. The carbonyl group υ(C=O) appears in the ligand at 1640 cm^−1^. In addition, the υ(S-H) bond stretching vibration band at 2830 cm^−1^ was observed, which demonstrates the solid-state formation of the thiol tautomer. The formation of the 1,3,4-oxadiazole ring was confirmed by the presence of new symmetric and asymmetric υ(C-O-C) stretching vibration bands characteristic of the ether group at approximately 1250 cm^−1^ and 1120 cm^−1^. In the ^1^H NMR spectrum (Figure S2), a singlet at 3.21 ppm is observed that integrates for two protons, H-11 and H-12; a multiplet at 7.30 ppm is observed that integrates for two protons, H-5 and H-6; a triplet at 7.51 ppm assigned to the proton H-4; and a double doublet at 7.71 ppm is observed that integrates for the proton H-3. Finally, at 12.41 ppm and 14.28 ppm, two singlets assigned to the protons of the imidazole ring H-1 and the oxadizolic ring H-18, respectively, are observed. In the mass spectrum of L, a peak at *m/z* = 274 is observed, indicative of the [M]^+^ ion.

The metallic derivative of BZ/OZ was further characterized using elemental analysis (C, N, H, and S), complexometric determination of metal concentration, IR spectroscopy, and the molar conductivity assessment. The metal complex was obtained as a beige solid soluble in DMF and DMSO and partially soluble in water, methanol, ethanol, chloroform and acetone. The findings from the elemental analysis and complexometry indicate a 2:1 ratio (ligand/metal). The molar conductivity in DMSO at 25 °C (207 Ω^−1^∙cm^2^∙mol^−1^) indicates the electrolytic nature of the complex [61]. Figure S1 displays the comparative FT-IR spectra of the precursor, the hybrid BZ/OZ ligand (L), and the BZ/OZ metallic derivative. Shifts in the bands corresponding to the vibrations υ(C=N), υ(C=O) and υ(C-O-C) corroborate the formation of the samarium complex (dashed lines presented in Figure S1). The metal complex exhibited chemical changes in the principal bands, suggesting coordination of the ligand and the metal center. The hypsochromic shifts from 1640 cm^−1^ to 1665 cm^−1^ and from 1120 cm^−1^ to 1200 cm^−1^ of the bands associated with the stretching vibrations of the C=O and C-O-C groups, respectively, along with the bathochromic shift in the stretching vibration band of the υ(C=N) bond [49], suggesting that the hybrid ligand BZ/OZ functions as a tridentate chelating agent via the carbonyl group and the nitrogen and oxygen atoms of the benzimidazole and oxadiazole rings, respectively. Although the stretching vibration band is red-shifted in the formation of many coordination complexes with carbonyl groups, the hypsochromic shift observed in the samarium complex is attributed to the coordination of the carbonyl oxygen to the Sm^3+^ cation (a severe Lewis acid), which decreases the electron density of oxygen and reduces the resonant participation of carbon, increasing the C=O double bond character [62]. The absence of significant π-backdonation by the metal center explains the shift direction [63].

### 3.2. Antibacterial Activity

In vitro antibacterial activity against Gram-positive bacteria (*Bacillus subtilis* ATCC 6633 and *Staphylococcus aureus* ATCC 6538) and Gram-negative bacteria (*Escherichia coli* ATCC 25922, *Klebsiella pneumoniae* ATCC 1705, *Klebsiella pneumoniae* ATCC 2146 and clinical strains *Shigella dysenteriae* and *Salmonella* spp.) were tested on L and its complex [SmL_2_]Cl_3_ by using the broth microdilution method. The minimum inhibitory concentrations (MICs) expressed in µg/mL were validated over three experiments and are listed in Table 1.

The MICs of L and [SmL_2_]Cl_3_ ranged from 250 to 1000 µg∙mL^−1^, being considerably higher compared to those of the reference antibiotics used in this study. The evaluated compounds demonstrated a predominant antimicrobial activity against Gram-negative bacterial strains. In particular, the ligand L inhibited the growth of all strains evaluated at a concentration of 1000 µg∙mL^−1^. These findings suggest that the metal derivative of the BZ/OZ ligand exhibits a higher antibacterial potency than the free BZ/OZ ligand, which could be explained through the overtone effect and the tweedy chelation theory [64]. According to the latter, in coordination complexes, the polarity of the metal ion is reduced and the lipophilicity of the complex increases, facilitating its interaction with the lipid membranes of pathogenic microorganisms [65], which is not the case with the starting samarium salt (SmCl_3_·6H_2_O), thus confirming the chelation theory. This interaction could contribute to the inhibition of enzymatic catalysis and/or DNA synthesis. The most sensitive bacterial strains were *Escherichia coli* ATCC 25922, *Klebsiella pneumoniae* ATCC 2146 and *Shigella dysenteriae*, with an MIC of 250 µg∙mL^−1^ for the Sm-complex. In contrast, the most resistant strains were *Bacillus subtilis* ATCC 6633, *Staphylococcus aureus* ATCC 6538 and *Salmonella* spp., which presented an MIC of 500 µg∙mL^−1^.

The antibacterial activity results are supported by additional studies performed on DMPC, DMPG and POPE membrane models (Section 3.3, Section 3.4 and Section 3.5) present in bacteria, which correlate with the antibacterial activity results of the metal complex [SmL_2_]Cl_3_, which showed significantly higher efficacy than the free ligand (L). This improvement in activity can be partly explained by biophysical studies that showed a higher interaction of the metal complex with the model membranes, especially those composed of phospholipids characteristic of bacteria such as DMPG and POPE [58,66]. Differential scanning calorimetry (DSC) studies showed that [SmL_2_]Cl_3_ significantly decreased the cooperativity of phase transitions and broadened their thermal range, indicating the formation of heterogeneous domains within the bilayer [67]. Consistently, Fourier transform infrared spectroscopy (FTIR) revealed shifts toward higher wavenumbers in the stretching bands of the -CH_2_ and C=O groups, which correlate with increased conformational disorder in the acyl chains and dehydration of the membrane interfacial region [68].

X-ray diffraction (SAXD and WAXD) analyses supported these findings showing an expansion of the interlamellar distance (d) and the water layer thickness (d_W_) in the presence of [SmL_2_]Cl_3_, particularly in DMPC-based systems [69]. Despite these alterations, the lamellar organization was maintained, suggesting that the complex does not destroy the bilayer but rather modifies its internal organization and hydration; a phenomenon that could promote permeabilization without the need for complete lysis [54,69]. In contrast, the free ligand L showed milder biophysical effects, consistent with its lower antimicrobial activity [67]. These modifications in membrane structure and dynamics suggest a non-lytic membranolytic mechanism of action or selective structural destabilization; a phenomenon previously reported for compounds with amphiphilic properties or high lipophilicity induced by metal complexation [70].

### 3.3. Membrane DSC Studies

**Figure 2:**
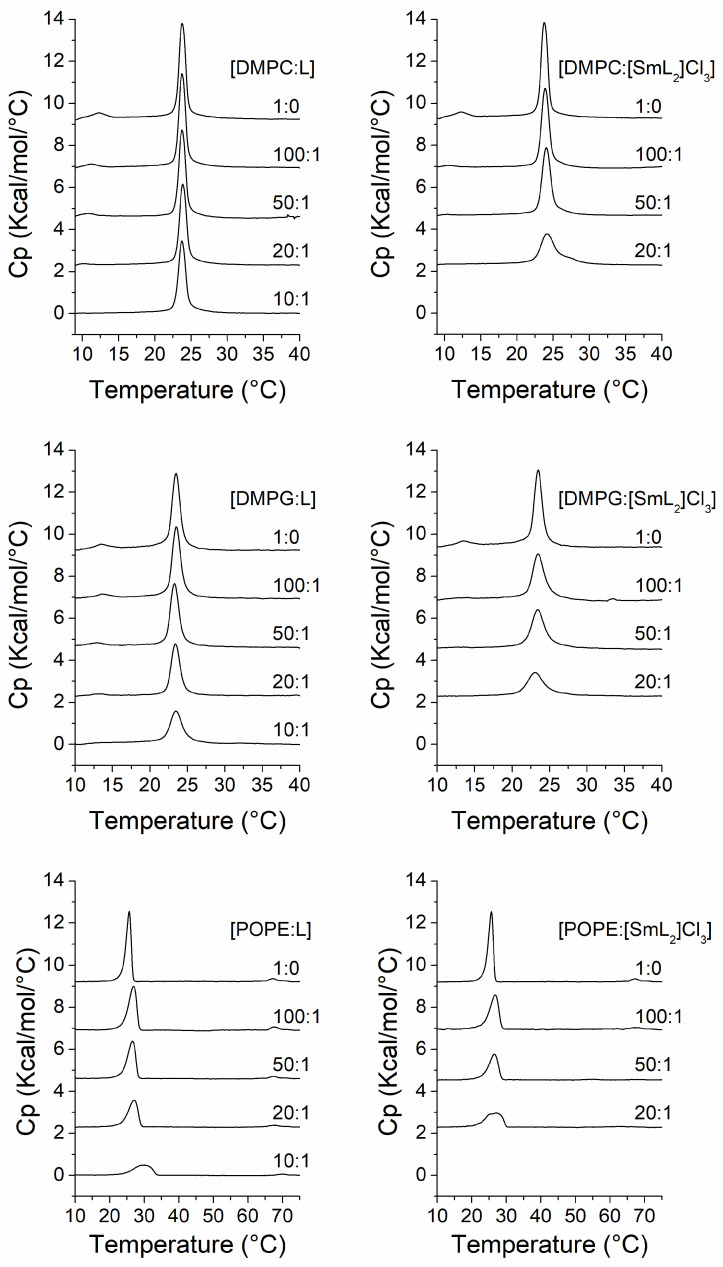
DSC thermograms of DMPC (**top panels**), DMPG (**middle panels**) and POPE (**bottom panels**) containing different concentration of L (**left**) and [SmL_2_]Cl_3_ (**right**). Compound molar ratios are displayed on the traces.

In phospholipid systems, differential scanning calorimetry allows the precise observation of thermotropic phase transitions and their modulation by the effect of compounds added to the system [71]. Pure DMPC, DMPG and POPE showed a polymorphic behavior with a highly cooperative endothermal phase transition from gel state (Lβ) to liquid-crystalline (Lα) state at temperatures of 23.7, 23.5 and 25.6 °C, respectively. DMPC and DMPG also showed a pretransition from the gel (Lβ’) to ripple phase (Pβ’); the former at a temperature of 12.2 °C, and the latter at 13.5 °C. Otherwise, POPE exhibited a transition from lamellar to hexagonal H_II_ phase at approximately 67 °C (Figure 2).

In the case of DMPC (Figure 2, top panels), ligand BZ/OZ (L) did not particularly affect the temperature of the main transition even at the highest concentration (10:1 molar ratio) of the compound, while the peak of the pretransition started to decrease in both temperature and intensity from the 100:1 DMPC/L molar ratio until it totally disappeared at a 10:1 molar ratio. The effect on the pretransition is a characteristic of intrinsic molecules. Conversely, the presence of [SmL_2_]Cl_3_ resulted in a more evident change in the temperature of the main transition, producing a widening of the peak, and thus exhibiting a decrease in the cooperativity of the transition, which is inversely proportional to the width of the transition peak. In general, this phenomenon occurs when a compound is incorporated into the bilayers interacting with the phospholipid, it decreases the cooperativity of the gel to liquid-crystalline phase transition, resulting in domains within the membrane with different transition temperatures [72]. In this case, at the concentration of 20:1 DMPC/[SmL_2_]Cl_3_ molar ratio, causes a decrease of the temperature of the onset of transition from 22.5 in the absence of [SmL_2_]Cl_3_ to 21 °C, and an increase of the temperature of the end of transition from 24.9 to 28.8 °C (Table 2), clearly indicating a reduction in cooperativity. Moreover, [SmL_2_]Cl_3_ also influenced the pretransition, which disappeared at the lowest concentration used (100:1 molar ratio).

Regarding the transition temperatures, in the DMPG model membrane a behavior like that of DMPC was observed. In fact, ligand L did not affect the temperatures at the onset and end of the main transition and only at the highest concentration exhibited some changes (Figure 2, middle panels, and Table 2), whilst the peak due to the pretransition gradually decreased until disappearance without any transition temperature changes. In this system, it was observed that complex [SmL_2_]Cl_3_ had a similar effect to that observed on DMPC, as the pretransition disappeared at the lowest concentration used and the transition from gel to liquid-crystalline state, as was the case on DMPC, indicated that the cooperativity gradually decreased as the [SmL_2_]Cl_3_ concentration increased.

Finally, as shown in the bottom panels of Figure 2, the thermogram profiles of POPE, in the presence of different concentrations of L and [SmL_2_]Cl_3_, changed. It is noteworthy that at higher concentrations of the compounds a broadening and an upshift in the temperature of the main transition were observed. In this system, even a slight increase in concentration of L or [SmL_2_]Cl_3_ induced a progressive loss of cooperativity in the phase transition; this being particularly evident in the 10:1 lipid/L molar ratio sample, with a considerable increase in temperature at the end of the transition from 26.7 °C in the absence of L to 33.5 °C (Table 2). POPE has no pretransition but has a second transition from the lamellar to hexagonal H_II_ phase at a higher temperature than that of the main transition. The presence of L did not affect this second transition even at higher concentrations. Conversely, the effect of [SmL_2_]Cl_3_ on the lamellar to hexagonal H_II_ phase transition was more drastic, resulting in the disappearance of the transition starting from the 50:1 lipid/[SmL_2_]Cl_3_ molar ratio, indicating that [SmL_2_]Cl_3_ favors the negative curvature of the membrane.

Concerning the enthalpy associated with the transition from gel to liquid-crystalline, a gradual decrease was recorded with both compounds in all model membranes, even when the effect of L on the aqueous dispersion of DMPC was lower than that of the other systems, with the maximum decrease in enthalpy being 0.86 Kcal/mol at the highest concentration of L. Alterations in the phase transition enthalpy are due to the interposition of the compounds between the acyl chains of phospholipids altering the packing of these chains by inducing disorder [73]. All enthalpies associated with the main transition of the different model membranes at all concentrations of the compounds are reported in Table 3.

**Figure 3:**
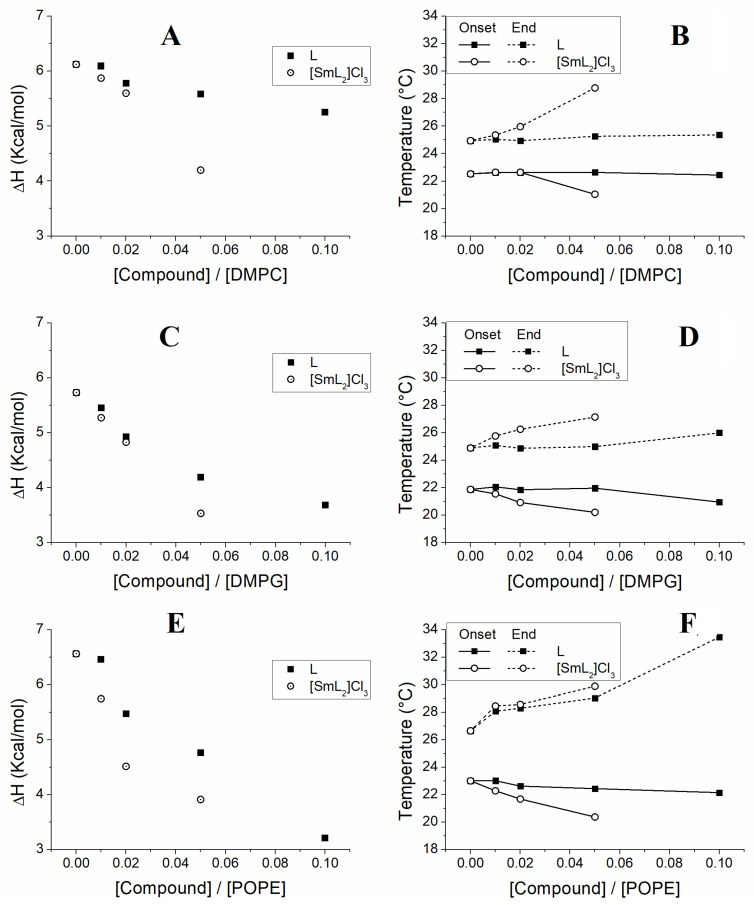
ΔH values versus molar fractions of L (■) and [SmL_2_]Cl_3_ (○) are depicted in the panels (**A**) (DMPC), (**C**) (DMPG) and (**E**) (POPE). Temperatures at the onset (continuous lines) and end (dotted lines) of the main phase transition are shown in the panels (**B**) (DMPC), (**D**) (DMPG) and (**F**) (POPE). The [compound]/[lipid] molar fraction values of 0, 0.01, 0.02, 0.05 and 0.1 correspond to lipid/compound molar ratios of 1:0, 100:1, 50:1, 20:1 and 10:1, respectively (as also reported in Table 2 and Table 3).

The values of enthalpies from Table 3 were plotted against the concentrations exhibiting the effect of the compounds on the membranes (Figure 3A,C,E). In addition, by using the temperatures obtained from the DSC scans (Figure 2, Table 2), a partial phase diagram was constructed for the phospholipid component (Figure 3B,D,F). This diagram indicates that by increasing the concentration of the compounds, a non-linear decrease in temperature is produced. We are in a situation where there is an immiscible phase, which would be an immiscible fluid phase in the case of the temperatures at the end of the transition or an immiscible gel phase in the case of the temperatures at the onset of the transition. On the contrary, in our situation the changes in all cases occurred in a fairly linear manner indicating a homogeneity of the membrane with a good miscibility of the lipids.

The dependence with temperature of both the ΔH and the temperatures at the onset and end of the main transition is shown in Figure 3, where it is clearly observed that L did not have much influence on either ΔH (Figure 3A) or the transition temperatures (Figure 3B) of DMPC, which remained almost unchanged at all concentrations of L. Vice versa, for [SmL_2_]Cl_3_ a marked change was observed, but only at a 20:1 DMPC/[SmL_2_]Cl_3_ molar ratio. In the DMPG model membranes, both L and [SmL_2_]Cl_3_ induced a decrease in enthalpy even at lower concentrations, which occurred progressively as the concentration of the compounds increased (Figure 3C). Inversely, as shown in Figure 3D, while the incorporation of [SmL_2_]Cl_3_ also produced a quite evident change in the onset and end temperatures of the transition, L modified these parameters only at the highest concentration. However, the greatest effect of the two compounds was observed on the POPE model membranes, where their presence induced both a significant decrease in enthalpy (Figure 3E), being this particularly evident in the case of [SmL_2_]Cl_3_, and a progressive increase in the temperature at the main transition’s end. Otherwise, for the temperature at the transition’s onset only a decrease in POPE/[SmL_2_]Cl_3_ was recorded, while remaining almost constant in the POPE/L system (Figure 3F).

### 3.4. Membrane FTIR Studies

The application of FTIR spectroscopy for the study of phase behavior of phospholipid membranes allows the monitoring of several functional groups to obtain information about lipid–lipid interactions at the molecular level [66]. The two most important groups of vibrational modes are the acyl chain and polar head vibrational modes. The most intense bands in the region of the acyl chains are generally the antisymmetric stretching band and the symmetric stretching band of the -CH_2_ groups, which appear around 2920 and 2850 cm^−1^, respectively. On the other hand, in the region between 1750 and 1700 cm^−1^, the most characteristic infrared band of the polar heads, the tension band of the C=O groups, appears [66]. The transition of the main phase of hydrated membranes from gel to liquid-crystalline implies important structural changes with the introduction of a conformational disorder (*gauche* conformers) that is observed in the infrared spectrum. Once the transition occurs, there is an intense effect on the wavenumber of the -CH_2_ and C=O stretching bands that can be correlated with an increase in the population of gauche conformer of the phospholipid in the liquid-crystalline phase.

**Figure 4:**
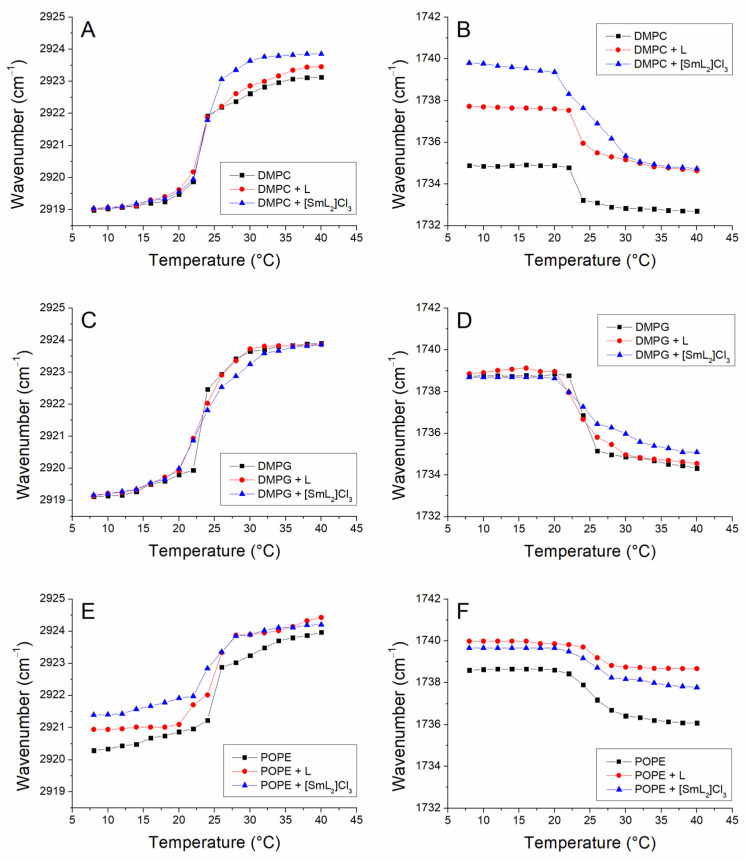
Temperature dependence of the maximum frequency of the absorbance antisymmetric stretching band of the -CH_2_ (left panels) and C=O (right panels) groups of DMPC (**A**,**B**), DMPG (**C**,**D**) and POPE (**E**,**F**). (■) are pure lipids curves, (●) are lipids/L (10:1 molar ratio) curves and (▲) are lipids/[SmL_2_]Cl_3_ (20:1 molar ratio) curves.

Figure 4 shows the temperature dependence of the antisymmetric stretching band of the -CH_2_ groups in the DMPC (panel A), DMPG (panel C) and POPE (panel E) model membranes as well as the effect of the incorporation of the compounds. The results obtained with both the symmetric and the antisymmetric bands were similar, but for our purposes the most evident antisymmetric tension band was chosen. The temperature dependence of the carbonyl band is represented in panels B (DMPC), D (DMPG) and F (POPE) of the same figure. For pure DMPC in the gel phase the maximum value of the antisymmetric stretching -CH_2_ band was around 2919 cm^−1^ and remained constant until the phase transition temperature from gel to liquid-crystalline (24 °C), where an abrupt shift in wavenumbers was observed, reaching values of more than 2923 cm^−1^, i.e., a wavenumber increment of around 4 cm^−1^. The incorporation of L and [SmL_2_]Cl_3_ in 10:1 and 20:1 DMPC/compound molar ratios did not produce important changes in the phase transition and in its transition temperature (Table 2). These results are in line with those previously obtained by differential scanning calorimetry, where none of the two compounds L or [SmL_2_]Cl_3_ affected the DMPC transition temperature. Although, it was interesting to observe that the incorporation of these amounts of L and, in particular, of [SmL_2_]Cl_3_ in DMPC vesicles shifted the stretching bands of the -CH_2_ groups to higher wavenumber values above the phase transition. This indicates that in the liquid-crystalline phase of the phospholipid, the compounds caused an additional disordering effect on the movement of the acylic chains and considerably increased the number of gauche conformers. Figure 4B shows the maximum values of the C=O bands of pure DMPC and of DMPC in the presence of the compounds L and [SmL_2_]Cl_3_. This is one of the most useful infrared bands, corresponding to the vibrational modes of the polar head region of the phospholipids and permitting the monitoring of changes in the interfacial region of the membrane [66]. From the pure DMPC graph, it could be seen that the gel to liquid-crystalline phase transition of the phospholipid was also reflected in the C=O stretching, with an acute change in wavenumber from 22 °C, due to an increase in the hydration of the polar heads of the phospholipid after the main phase transition, which was calculated at a temperature of 23.4 °C (Table 4). The C=O groups of the ester bonds of phospholipids dispersed in water can establish a number of hydrogen bonds with the water molecules of the hydration layer surrounding the bilayer; this is what is commonly called C=O groups in the hydrated state [74]. The incorporation of the compounds did not generate any important effects in the case of L, while the presence of [SmL_2_]Cl_3_ caused the onset of the transition to move to lower temperatures, the end shifted to higher temperature values, indicating a decrease in cooperation, similarly to what is observed in the DSC data shown above. In addition, the Tm showed a small increase due to the presence of [SmL_2_]Cl_3_. However, the main effect of the compounds was to produce a strong shift in the band maximum towards higher wavenumbers (about 5 cm^−1^ for [SmL_2_]Cl_3_ for example), both above and below the transition temperature. These shifts towards higher wavenumbers were attributed to the interaction of L and [SmL_2_]Cl_3_ with DMPC causing a strong dehydration of the carbonyl groups of the phospholipid, and therefore, of the interfacial region of the membrane.

In the case of DMPG, the examination of the antisymmetric stretching band of the -CH_2_ groups indicated the absence of any effect of both compounds at the maximum of this band, both above and below the phase transition temperature (Figure 4C). Here, only a slight decrease in the cooperation could be appreciated, especially due to the incorporation of [SmL_2_]Cl_3_, since the Tm did not undergo any kind of change. The representation of the maximum band of C=O vs. temperature, shown in Figure 4D, besides confirming a slight decrease in cooperativity due to the insertion of the compounds, also showed a decrease in hydration in the polar part of the DMPG membrane caused by the presence of [SmL_2_]Cl_3_.

Finally, Figure 4E,F shows, the dependence of both the acyclic and carbonyl groups on the temperature in the model membranes of POPE and the impact of the insertion of L and [SmL_2_]Cl_3_, respectively. In this system, as observed with DSC, the largest effect of the two compounds was detected. Concerning the transition temperature calculated from the representation of the band of the -CH_2_ group, both compounds induced a small decrease that was not observed in the graph of the carbonyl group vs. temperature. It was instead evident that both L and [SmL_2_]Cl_3_, induced a progressive expansion of the transition, reflecting a decrease in cooperativity and inducing a shift towards higher wavenumbers of the -CH_2_ band, both below and above the transition temperature. The two compounds also showed a remarkable effect on the band associated with the C=O group, inducing a higher dehydration of the interface of the POPE membrane in the gel phase that is maintained even after the transition. In this case, a stronger effect was observed, caused by L, unlike the effect on acylic chains which was more evident with [SmL_2_]Cl_3_. The Tm of all model membranes used in the absence and in the presence of L and [SmL_2_]Cl_3_ calculated by the stretching bands of both the -CH_2_ and C=O groups, are shown in Table 4.

### 3.5. Membrane X-Ray Studies

**Figure 5:**
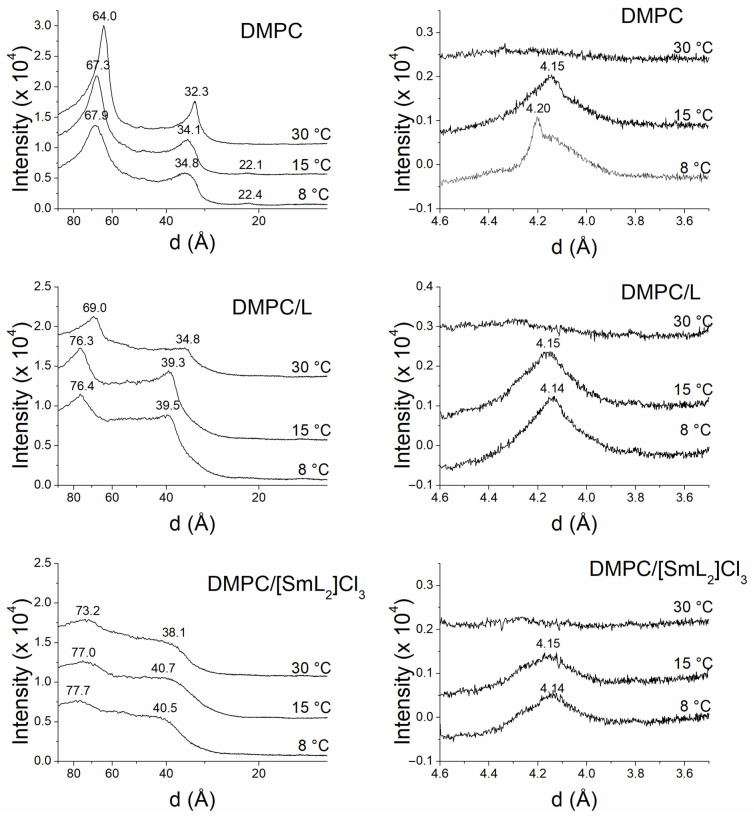
Small- and wide-angle X-ray diffractograms of pure DMPC (**top panel**), lipid/L (**middle panel**) and lipid/[SmL_2_]Cl_3_ (**bottom panel**). Molar ratios were 10:1 phospholipid/L and 20:1 phospholipid/[SmL_2_]Cl_3_. Temperatures are shown on the diffractograms.

**Figure 6:**
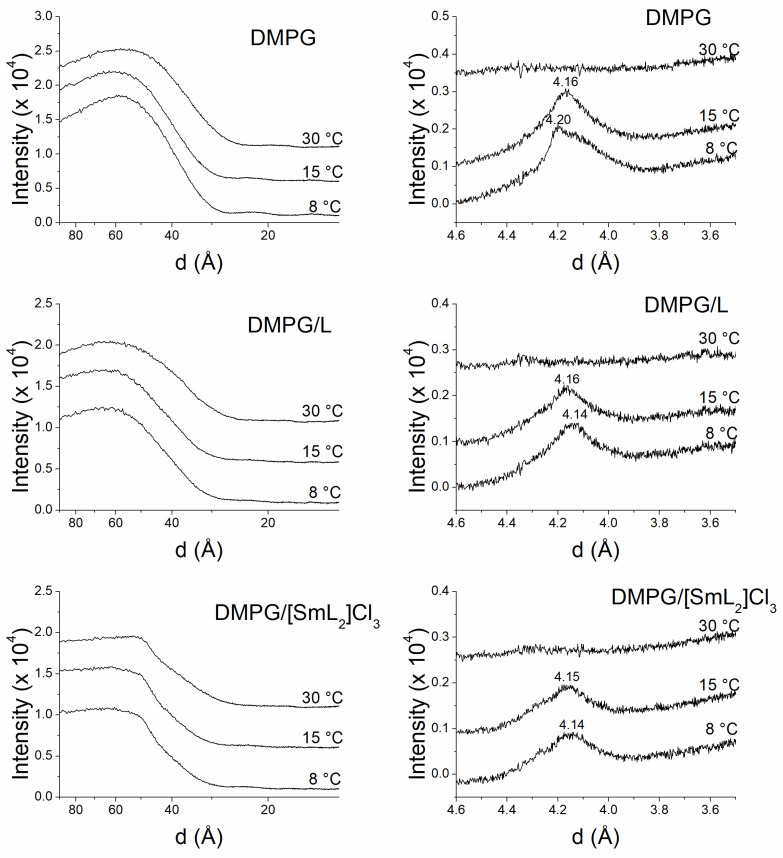
Small- and wide-angle X-ray diffractograms of pure DMPG (**top panel**), lipid/L (**middle panel**) and lipid/[SmL_2_]Cl_3_ (**bottom panel**). Molar ratios were 10:1 phospholipid/L and 20:1 phospholipid/[SmL_2_]Cl_3_. Temperatures are shown on the diffractograms.

**Figure 7:**
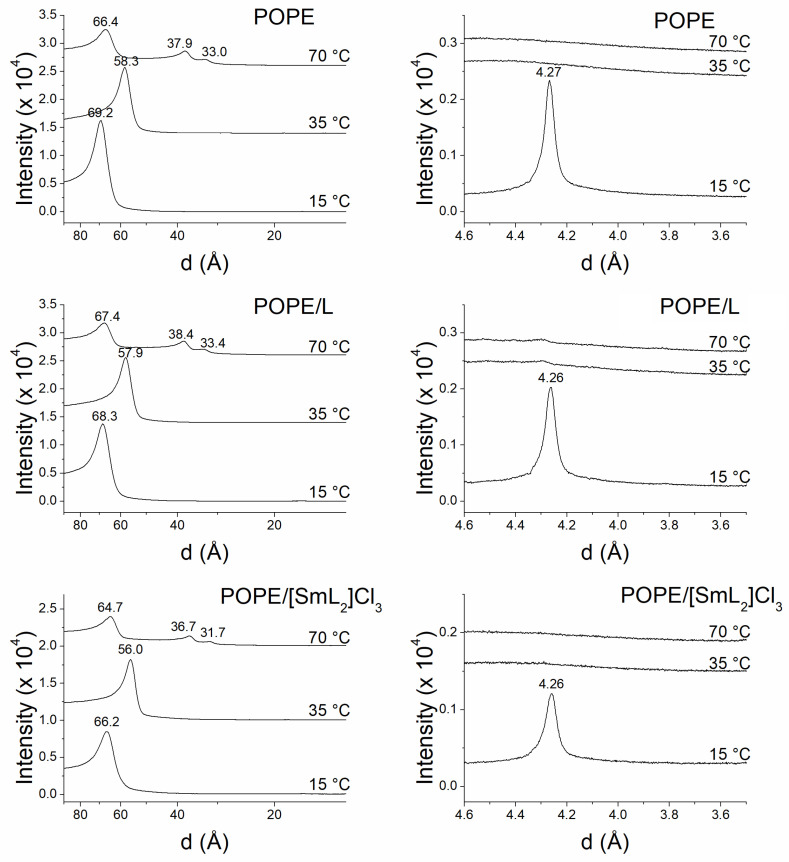
Small- and wide-angle X-ray diffractograms of pure POPE (**top panel**), lipid/L (**middle panel**) and lipid/[SmL_2_]Cl_3_ (**bottom panel**). Molar ratios were 10:1 phospholipid/L and 20:1 phospholipid/[SmL_2_]Cl_3_. Temperatures are shown on the diffractograms.

The analysis of the information obtained by SAXD not only defines the macroscopic structure but also provides the interlamellar repetition distance of the lamellar phase. The most intense first-order reflection corresponds to the width of the bilayer plus the water layer between bilayers [75]. On the other hand, the data for the WAXD region provides information about the packing of the acylic chains of the phospholipids [69]. Figure 5 and Figure 6 show the SAXD and WAXD diffraction patterns of DMPC and DMPG, respectively, at 8, 15 and 30 °C, while Figure 7 shows the diffraction patterns of POPE at 15, 35 and 70 °C in the absence and in the presence of L and [SmL_2_]Cl_3_. The temperatures were chosen to analyze phospholipids in the different thermotropic phases adopted by each of them according to the data obtained from the DSC experiments. For DMPC and DMPG the Lβ’, Pβ’ and Lα phases that these phospholipids assume at 8, 15 and 30 °C were studied, while for POPE the studies where performed for Lβ, Lα and H_II_ phases at 15, 35 and 70 °C, respectively.

In the case of DMPC, the SAXD patterns exhibited acute Bragg reflections (d-spacing), related as 1:1/2:1/3, at the lowest temperatures and 1:1/2 at 30 °C, indicating the existence of multilamellar systems [76] with an interlamellar repetition distance d of 67.9 Å to 8 °C, 67.3 Å to 15 °C and 64.0 Å to 30 °C.

Figure 5 also displays the DMPC WAXD profiles obtained at the same temperatures. At 8 °C, it was possible to detect a net reflection at 4.20 Å and a wide shoulder around 4.10 Å, which is indicative of a gel Lβ’ phase with the lateral chains of hydrocarbons inclined with respect to what is normal for the bilayer phase and a pseudohexagonal chain packing [68]. At 15 °C, however, the wide spacing centered at 4.15 Å indicated that the phase was not Lβ’ but a ripple Pβ’ phase, since this temperature is above the pretransition, although still below the main transition. Finally, at 30 °C, a diffuse WAXD reflection typical of unordered lipid chains in the liquid-crystalline Lα phase was observed.

The incorporation of L and [SmL_2_]Cl_3_ induced disorder in the DMPC membrane structure, due to a greater diffusion and broadening of d-spacing, which was more evident in the presence of [SmL_2_]Cl_3_. However, the presence of only two reflections related as 1:1/2 indicated a good homogeneity in the distribution of both compounds incorporated into the membrane and that the bilayer organization was still multilamellar. Moreover, an increase in the interlamellar repetition distance was also observed, being d = 76.4 Å at 8 °C, 76.3 Å at 15 °C and 69.0 Å at 30 °C in presence of L, and d = 77.7 Å at 8 °C, 77 Å at 15 °C and 73.2 Å at 30 °C with [SmL_2_]Cl_3_. These d-spacing values suggested that even for the interlamellar repetition distance, [SmL_2_]Cl_3_ had a slightly greater effect than L especially in the Lα phase. The profiles of WAXD exhibited a wide peak centered at 4.14 Å at 8 °C and at 4.15 Å at 15 °C, both with L and [SmL_2_]Cl_3_, confirming the absence of the pretransition observed by DSC and indicating that below the transition temperature the two compounds at the concentrations used prevented the formation of the Lα’ phase present in the membrane of pure DMPC. On the other hand, at 30 °C the wide-angle patterns of the DMPC samples with L and [SmL_2_]Cl_3_ exhibited a diffuse reflection typical of disordered acyclic chains in a liquid-crystalline phase like that of pure DMPC.

In the case of DMPG (Figure 6), SAXD profiles in the absence and presence of L and [SmL_2_]Cl_3_ exhibited diffuse scattering at all temperatures studied with a total lack of Bragg diffraction observed for DMPC. This type of pattern is typical of a single bilayer not positionally correlated, probably due to the negative charge of the membrane surface of DMPG that generates a difference in electron density between the inside of the bilayer and the aqueous solvent [77]. And likely because of that, DMPG membranes could be arranged in large unilamellar vesicles or unorganized multilayers [78,79]. However, the incorporation of the two compounds did not alter the organization of the phospholipids, although the patterns showed a slightly different appearance. Due to these characteristics of the diffraction profiles, it was impossible to determine the interlamellar repetition distance d. On the other hand, in the absence and presence of the two compounds, DMPG WAXD scattering patterns were like those of DMPC. In fact, pure DMPG exhibited the typical diffraction profiles of a gel Lβ’ phase at 8 °C, indicated by the presence of a reflection peak at 4.20 Å and a shoulder at about 4.10 Å, a ripple Pβ’ phase at 15 °C, as after the pretransition the shoulder disappeared and the peak centered at 4.16 Å, and finally a liquid-crystalline Lα phase at 30 °C, where the disordered lipid chains generated a typical diffuse reflection. The presence of the two compounds induced, as was the case for DMPC, the lack of pretransition, leading to similar diffraction profiles at 8 and 15 °C typical for the existence of a gel Lβ phase. At 30 °C, in both cases a diffuse reflection was observed indicating the presence of a lamellar Lα phase [80].

In pure POPE SAXD patterns (Figure 7), only a single lamellar reflection was observed at 15 and 35 °C temperatures, i.e., below and above the gel to liquid-crystalline phase transition, indicating the presence of a lamellar phase [81]. On the other hand, at 70 °C, POPE samples showed three reflections that were related as 1:1/√3:1/√4, corresponding to the proportion of the periodicity parameter due to an inverted hexagonal H_II_ phase [82]. The addition of L and [SmL_2_]Cl_3_ did not perturb the lamellar organization of the phospholipid, neither below nor above the gel to liquid-crystalline phase transition and neither of the inverted hexagonal H_II_ phase. Nevertheless, while L apparently did not alter even the interlamellar repetition distance, there was a slight decrease in d-spacing in the case of [SmL_2_]Cl_3_. The values of d observed were: 69.2 Å (POPE), 68.3 Å (with L) and 66.2 Å (with [SmL_2_]Cl_3_) at 15 °C; 58.3 Å (POPE), 57.9 Å (with L) and 56.0 Å (with [SmL_2_]Cl_3_) at 35 °C; and in the hexagonal phase 66.4 Å (POPE), 67.4 Å (with L) and 64.7 Å (with [SmL_2_]Cl_3_) at 70 °C. Concerning the WAXD patterns, none of the two compounds induced any kind of change to the profiles observed for pure POPE, which showed the typical characteristics of Lβ at 15 °C, and Lα at 35 and 70 °C.

**Figure 8:**
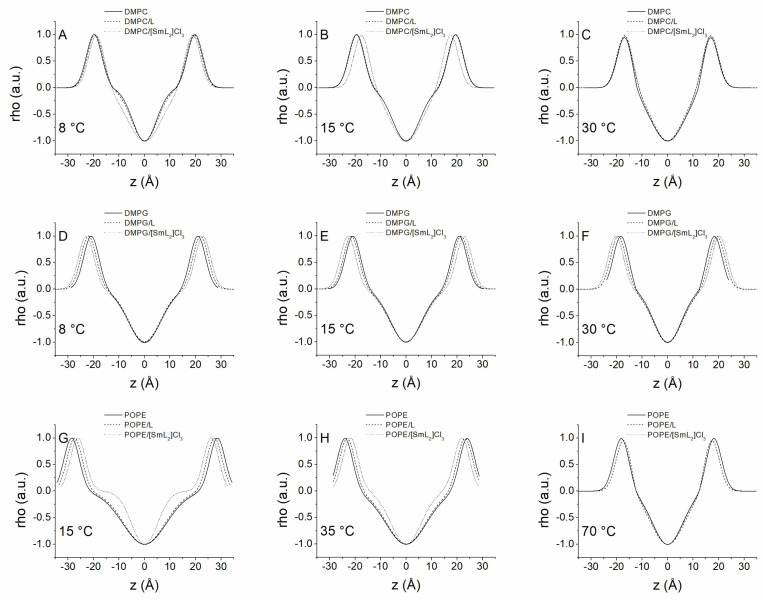
One-dimensional electron density profiles calculated from SAXD profiles of (**A**) DMPC at 8 °C; (**B**) DMPC at 15 °C; (**C**) DMPC at 30 °C; (**D**) DMPG at 8 °C; (**E**) DMPG at 15 °C; (**F**) DMPG at 30 °C; (**G**) POPE at 15 °C; (**H**) POPE at 35 °C; (**I**) POPE at 70 °C, in the presence and absence of L (10:1 molar ratio) and [SmL_2_]Cl_3_ (20:1 molar ratio).

To further study the structure of the bilayers and the effect of the compounds on them, the SAXD patterns of all systems were analyzed using the Global Analysis Program (GAP). Figure 8 shows the corresponding one-dimensional electron density profiles due to the bilayers being perpendicular, calculated from the SAXD patterns shown in Figure 5, Figure 6 and Figure 7. With this method it was possible to calculate different structural parameters derived from the electronic density profiles (Table 5). Given the good miscibility shown by both compounds at the concentrations employed, from which homogeneous structures were derived at all temperatures studied, this method was applied to all diffractograms. The d values shown in Table 5 were calculated with the GAP software version 1.3 in such a way that it is possible to detect very small differences with the d-spacing values observed directly from the SAXD diffraction profiles, although they remain within the experimental error range. In general, in all pure phospholipid systems employed, a decrease in membrane thickness d_B_ was observed after the phase transition from gel to liquid-crystalline, which was a consequence of the increase in lipid disorder. In the case of DMPC, the incorporation of L did not lead to significant changes in hydrophobic membrane thickness at any temperature, while [SmL_2_]Cl_3_ induced a decrease in d_B_ associated with the formation of phase P’. However, the factor that incremented the interlamellar thickness, observed after the insertion of both compounds, was a marked increase of d_W_, indicating an expansion of the water layer thickness of 10 or more Å, irrespective of the phase in which the membrane was. Through the GAP, we were able to calculate the membrane thickness for DMPG samples as well. However, since they are not form-ordered multilamellar structures, it was impossible to obtain other parameters. In addition, it was observed that above the transition temperature d_B_ decreased, but unlike DMPC, the presence of L and [SmL_2_]Cl_3_ resulted in an increase in membrane thickness, which was particularly evident for [SmL_2_]Cl_3_. This is possibly due to a rigidity of the membrane caused by the presence of the compounds.

Finally, regarding POPE, it is noteworthy that the distance between phosphate groups, and consequently d_B_, dropped dramatically as the temperature increased. Clearly, these changes were due to the different organizations that POPE assumed, changing from the lamellar Lβ phase to the lamellar Lα phase and up to the inverted hexagonal H_II_ phase. In this lipid system, it is worth focusing on d_W_, which in the lamellar phase was almost zero, while it increased drastically when POPE passed to the hexagonal phase. These values may suggest that the lamellar phase is not organized in multilamellar vesicles, as was the case with DMPC, but that the hexagonal phase d_W_ is the width of the water inside the individual micelles. The incorporation of the two compounds did not modify the structural organization of the aqueous dispersion of POPE. However, the introduction of L into the membrane induced a decrease in d_B,_ in both the lamellar and hexagonal phases, while an increase of d_W_ was observed (Table 5). Inversely, with [SmL_2_]Cl_3_ the membrane thickness decreased in the lamellar phase but remained constant in the hexagonal phase. Finally, unlike L, [SmL_2_]Cl_3_ also generated a decrease in the water width of the H_II_ phase.

### 3.6. In Silico Toxicological Predictions

The results obtained through in silico predictions revealed significant toxicological differences among the three analyzed molecules, highlighting distinct potential risks and applications for each compound (Table 6). Ligand exhibited an intermediate toxicological profile, with positive mutagenicity in the Ames test (M+) and carcinogenicity specific to female mice (C+) but not to rats (C−). This selectivity may be related to metabolic differences between species, a phenomenon previously described in the literature [83]. Additionally, its classification as causing “mild” skin and ocular irritation suggests a moderate risk in dermal exposures, while its oral LD_50_ (0.013 g/kg) and EC_50_ in *Daphnia magna* (2.74 mg/L) indicate moderate acute toxicity. The TD_50_ value (48 mg/kg in mice and 22 mg/kg in rats) further underscores a carcinogenic potential that warrants attention in long-term exposure studies.

Sm(III) complex stood out negatively, with high carcinogenic potential (extremely low TD_50_: 0.01 mg/kg in mice and 0.0292 mg/kg in rats), as well as elevated acute toxicity (oral LD_50_ = 0.000418 g/kg; EC_50_ = 0.0178 mg/L). These findings suggest that even small doses may be sufficient to induce severe adverse effects, including carcinogenesis and chronic toxicity (LOAEL = 0.0826 g/kg). Despite being non-mutagenic (M−), its skin sensitization potential and mild ocular irritations reinforce the need for careful handling. These results align with studies linking specific chemical structures to high systemic toxicity indices. The results corroborate the utility of in silico tools, such as TOPKAT, in preliminary toxicological screening of compounds, enabling the rapid identification of priority risks [60]. The discrepancy among the molecular profiles emphasizes the importance of a multifactorial assessment, integrating parameters such as carcinogenicity, acute/chronic toxicity and irritation.

For pharmaceutical or industrial applications, Sm(III) complex would require extensive structural modifications to reduce its carcinogenicity and toxicity, whereas L could be reconsidered for specific uses, with rigorous monitoring of its carcinogenicity in mice. In vitro and in vivo studies are recommended to validate these predictions, particularly for parameters showing the greatest interspecies discrepancies.

The in vitro biological activity results presented in this study showed that the metal complex [SmL_2_]Cl_3_ exhibits significantly higher antibacterial activity than the free ligand (Section 3.2). In contrast, although the free ligand L showed lower antimicrobial efficacy, its toxicological profile was more moderate, with specific but manageable risks. This discrepancy between efficacy and safety underscores the need to optimize the structure of the metal complex to reduce its toxicity without compromising its biological activity.

To address this challenge, several modifications in the structure of the ligand moiety could be necessary to increase the balance between improving solubility and reducing toxicity. A first approach involves the inclusion of electron-rich substituents or polar groups, such as hydroxyl and carboxyl, to improve aqueous solubility [60,84]. Furthermore, the incorporation of biocompatible scaffolds or biodegradable components can help further reduce long-term toxicity issues [60,85]. In addition, the introduction of nanoparticles [60,86,87] or liposomal carrier systems has been shown to provide greater solubility and increase target site specificity [88]. These structural changes are therefore of great importance in the design of new ligands that have potential for clinical applications with greater therapeutic power and fewer toxic side effects.

## 4. Conclusions

This study demonstrates that hybrid heteroaromatic compounds based on benzimidazole and oxadiazole (BZ/OZ), particularly in their complexed form with samarium ([SmL_2_]Cl_3_), exhibit mild antibacterial effects, displaying slightly higher activity against Gram-negative bacterial strains. The combination of microbiological assays and biophysical studies on membrane models revealed that this activity is closely associated with the ability of the metal complex to alter the structural, thermotropic and interfacial properties of model lipid bilayers, without drastically affecting their lamellar organization. However, in silico toxicological prediction results indicate that the samarium complex has high toxicity and carcinogenic potential that limits its therapeutic capacity, so structural modifications are necessary. Introducing hydroxyl or carboxylate groups in the ligand structure could increase its hydrophilicity and chelating capacity, favoring a partial Sm–O type coordination that decreases the effective positive charge density of the metal, thus reducing non-selective penetration into cell membranes and stabilizing the complex in physiological media. Additionally, the partial substitution of chlorides by less coordinating counterions, such as nitrate or acetate, could soften the Lewis acidity of Sm(III) and attenuate its irritating and cytotoxic potential. In contrast, the free ligand (L) showed a more favorable toxicological profile, albeit with lower antimicrobial efficacy. These findings highlight the importance of integrating in vitro, in silico and biophysical studies for the rational design of antimicrobial agents and suggest future research should focus on optimizing the structure of metal complexes to achieve a balance between efficacy and safety.

## Figures and Tables

**Table 1:** MIC (µg∙mL^−1^) of BZ/OZ derivatives, L and [SmL_2_]Cl_3_.

Compound	Gram-Positive		Gram-Negative			
	*B. subtilis*	*S. aureus*	*E. coli*	*K. pneumoniae*	*S. dysenteriae*	*Salmonella*
L	1000	1000	1000	1000	1000	1000
[SmL_2_]Cl_3_	500	500	250	250	250	500
SmCl_3_·6H_2_O	>1000	>1000	>1000	>1000	>1000	>1000
Cp ^1^	<3.9	<3.9	<3.9	<3.9	<3.9	<3.9
AgNO_3_ ^1^	<7.8	<7.8	<7.8	<7.8	<7.8	<7.8

^1^ Drug control: Ciprofloxacin (Cp) and silver nitrate (AgNO_3_).

**Table 2:** Onset and end temperature of main phase transition of DMPC, DMPG and POPE dispersions in the absence and presence of L and [SmL_2_]Cl_3_ at different concentrations.

[Compound]/ [Lipid]	Molar Ratio (L:C)	DMPC/L		DMPC/[SmL_2_]Cl_3_		DMPG/L		DMPG/[SmL_2_]Cl_3_		POPE/L		POPE/[SmL_2_]Cl_3_	
		Onset	End	Onset	End	Onset	End	Onset	End	Onset	End	Onset	End
		(°C)											
0.00	1:0	22.5	24.9	22.5	24.9	21.9	24.9	21.9	24.9	23.0	26.7	23.0	26.7
0.01	100:1	22.6	25.0	22.6	25.4	22.1	25.1	21.5	25.8	23.0	28.1	22.3	28.5
0.02	50:1	22.6	24.9	22.6	26.0	21.9	24.9	20.9	26.3	22.6	28.3	21.7	28.6
0.05	20:1	22.6	25.3	21.0	28.8	22.0	25.0	20.2	27.1	22.4	29.0	20.4	29.9
0.10	10:1	22.4	25.4			21.0	26.0			22.2	33.5		

**Table 3:** Enthalpy (ΔH) of main phase transition of DMPC, DMPG and POPE dispersions in the absence and presence of L and [SmL_2_]Cl_3_ at different concentrations.

[Compound]/ [Lipid]	Molar Ratio (L:C)	DMPC/L	DMPC/[SmL_2_]Cl_3_	DMPG/L	DMPG/[SmL_2_]Cl_3_	POPE/L	POPE/[SmL_2_]Cl_3_
		ΔH (Kcal/mol)					
0.00	1:0	6.12	6.12	5.73	5.73	6.56	6.56
0.01	100:1	6.09	5.87	5.45	5.27	6.46	5.75
0.02	50:1	5.78	5.60	4.93	4.83	5.47	4.51
0.05	20:1	5.58	4.20	4.19	3.53	4.76	3.91
0.10	10:1	5.26		3.68		3.21	

**Table 4:** Main transition temperatures of DMPC, DMPG and POPE with and without L and [SmL_2_]Cl_3_, calculated by Boltzmann sigmoidal function using -CH_2_ and C=O stretching bands’ maximum positions. SD is the standard deviation.

	-CH_2_		C=O	
	Tm (°C)	SD	Tm (°C)	SD
DMPC	23.4	0.3	23.4	0.2
DMPC/L	23.5	0.3	23.9	0.3
DMPC/[SmL_2_]Cl_3_	23.7	0.2	25.2	0.2
DMPG	23.4	0.2	25.8	0.6
DMPG/L	23.2	0.1	24.0	0.2
DMPG/[SmL_2_]Cl_3_	23.5	0.2	25.2	0.4
POPE	25.3	0.4	25.5	0.2
POPE/L	24.7	0.3	25.5	0.2
POPE/[SmL_2_]Cl_3_	24.1	0.3	25.8	0.3

**Table 5:** Fitting parameters obtained by using the GAP. d, d-spacing; d_HH_, headgroup peak−peak distance; d_B_, total bilayer thickness (polar head plus hydrophobic layer); and d_W_, water layer.

	T (°C)	d (Å)	d_HH_ (Å)	d_B_ (Å)	d_W_ (Å)
DMPC	8	68.6	39.6	51.6	17.1
	15	67.8	39.0	51.0	16.8
	30	64.6	33.5	45.5	19.1
DMPC/L	8	78.0	38.9	50.9	27.2
	15	78.2	38.7	50.7	27.5
	30	70.4	33.8	45.8	24.7
DMPC/[SmL_2_]Cl_3_	8	79.5	37.7	49.7	29.8
	15	79.1	35.0	47.0	32.1
	30	73.8	33.2	45.2	28.5
DMPG	8		41.8	53.8	
	15		41.7	53.7	
	30		36.6	48.6	
DMPG/L	8		43.9	55.9	
	15		43.1	55.1	
	30		38.7	50.7	
DMPG/[SmL_2_]Cl_3_	8		45.7	57.7	
	15		45.5	57.5	
	30		40.6	52.6	
POPE	15	68.6	56.0	68.0	0.6
	35	57.4	45.1	57.1	0.3
	70	69.5	36.2	48.2	21.3
POPE/L	15	67.2	54.8	66.8	0.3
	35	57.4	44.6	56.6	0.8
	70	69.9	34.4	46.4	23.5
POPE/[SmL_2_]Cl_3_	15	65.2	52.2	64.2	0.9
	35	56.7	43.7	55.7	0.9
	70	66.8	36.2	48.2	18.6

**Table 6:** Computational toxicological parameters (CTP), prediction oral dose toxicity risk (PODTR) and tolerated dose of carcinogenic potency (TDCP) for BZ/OZ ligand and its Sm(III) complex *.

Molecule	CTP							PODTR				TDCP		
								OR		RC	RI	Mouse	Rat	Rat
	MF	MM	RF	AM	SI	SS	OI	EC_50_ (mg/L)	LD_50_ (g/kg)	LOAEL (g/kg)	LC_50_ (mg/m^3^/h)	TD_50_ (mg/kg)		RMTD (g/kg)
L	C+	C−	C−	M+	Mild	NS	Mild	2.74	0.013	0.201	2.55 × 10^3^	48	22	0.215
[SmL_2_]Cl_3_	C+	C−	C−	M−	NI	S	Mild	0.0178	0.000418	0.0826	42.6	0.01	0.0292	0.0169

* MF: Mouse Female; MM: Mouse Male; RF: Rat Female; AM: Ames Mutagenicity; SI: Skin Irritancy; NI: Nonirritant; SS: Skin Sensitization; S: Sensitizer; NS: Non Sensitizer; OI: Ocular Irritancy; OR: Oral Rate; LD_50_: Median lethal dose; FM: Fathead Minnow (Short-term toxicity to mouse); LC_50_: Exposure concentration of a toxic substance lethal to half of the animals tested; DM: Daphnia magna; EC_50_: Half maximal effective concentration (effective concentration of a substance causing adverse effects in 50% of the population tested-Daphnia magna); RC: Rat Chronic; LOAEL: lowest level of adverse effect observed; RI: Rat Inhalation; TD_50_: Carcinogenic potency value; RMTD: Rat Maximum Tolerated Dose; − (None); C (Single Carcinogen); C+ (Multi Carcinogen); C− (Non Carcinogen); M− (Non Mutagen); M+ (Mutagen); + (Weak).

## Data Availability

The original contributions presented in this study are included in the article/Supplementary Material. Further inquiries can be directed to the corresponding authors.

## Supplementary Materials

The following supporting information can be downloaded at: https://www.mdpi.com/article/10.3390/biom15111568/s1. Figure S1: FT-IR spectra of precursor hydrazide, ligand (L), and samarium complex; Figure S2: ^1^H NMR spectra of benzimidazole/oxadiazole ligand (L).

## References

[1] Ponzo E., De Gaetano S., Midiri A., Mancuso G., Giovanna P., Giuliana D., Zummo S., Biondo C. (2024). The Antimicrobial Resistance Pandemic Is Here: Implementation Challenges and the Need for the One Health Approach. Hygiene.

[2] Ahmed S.K., Hussein S., Qurbani K., Ibrahim R.H., Fareeq A., Mahmood K.A., Mohamed M.G. (2024). Antimicrobial Resistance: Impacts, Challenges, and Future Prospects. J. Med. Surg. Public Health.

[3] Salam M.A., Al-Amin M.Y., Salam M.T., Pawar J.S., Akhter N., Rabaan A.A., Alqumber M.A.A. (2023). Antimicrobial Resistance: A Growing Serious Threat for Global Public Health. Healthcare.

[4] Singh A.K., Kumar A., Singh H., Sonawane P., Paliwal H., Thareja S., Pathak P., Grishina M., Jaremko M., Emwas A.-H. (2022). Concept of Hybrid Drugs and Recent Advancements in Anticancer Hybrids. Pharmaceuticals.

[5] Sampath Kumar H.M., Herrmann L., Tsogoeva S.B. (2020). Structural Hybridization as a Facile Approach to New Drug Candidates. Bioorg. Med. Chem. Lett..

[6] Shabatina T.I., Vernaya O.I., Melnikov M.Y. (2023). Hybrid Nanosystems of Antibiotics with Metal Nanoparticles—Novel Antibacterial Agents. Molecules.

[7] Lungu I.-A., Moldovan O.-L., Biriș V., Rusu A. (2022). Fluoroquinolones Hybrid Molecules as Promising Antibacterial Agents in the Fight against Antibacterial Resistance. Pharmaceutics.

[8] Khwaza V., Oyedeji O.O., Morifi E., Nwamadi M., Fonkui T.Y., Ndinteh D.T., Aderibigbe B.A. (2025). Design and Synthesis of Hybrid Compounds for Potential Treatment of Bacterial Co-Infections: In Vitro Antibacterial and In Silico Studies. Antibiotics.

[9] Elwahy A.H.M., Hammad H.F., Ibrahim N.S., Al-Shamiri H.A.S., Darweesh A.F., Abdelhamid I.A. (2024). Synthesis and Antibacterial Activities of Novel Hybrid Molecules Based on Benzothiazole, Benzimidazole, Benzoxazole, and Pyrimidine Derivatives, Each Connected to N-Arylacetamide and Benzoate Groups. J. Mol. Struct..

[10] Ghods M., Almasirad A., Tahghighi A. (2025). Synthesis and in Vitro Anti-Bacterial Activity of Novel Quinoline-Based Aryl/Heteroaryl Amide Hybrids. J. Mol. Struct..

[11] Műller D., Krakowska A., Zontek-Wilkowska J., Paczosa-Bator B. (2025). Simple and Hybrid Materials for Antimicrobial Applications. Colloids Surf. B Biointerfaces.

[12] Lin Y., Betts H., Keller S., Cariou K., Gasser G. (2021). Recent developments of metal-based compounds against fungal pathogens. Chem. Soc. Rev..

[13] Al-Jameel S.S., Ababutain I.M., Alghamdi A.I., Ben-Ali A., Al-Nasir A.H., Alqhtani A.H., Aldewely L.K., Alhassan M.M., Bakhurji R.E., AlGhamdi W.M. (2024). Hybrid Organic-Inorganic Copper and Cobalt Complexes for Antimicrobial Potential Applications. Cell. Physiol. Biochem..

[14] Diaconu D., Mangalagiu V., Amariucai-Mantu D., Antoci V., Giuroiu C.L., Mangalagiu I.I. (2020). Hybrid Quinoline-Sulfonamide Complexes (M2+) Derivatives with Antimicrobial Activity. Molecules.

[15] Diaconu D., Antoci V., Mangalagiu V., Amariucai-Mantu D., Mangalagiu I.I. (2022). Quinoline–imidazole/benzimidazole derivatives as dual-/multi-targeting hybrids inhibitors with anticancer and antimicrobial activity. Sci. Rep..

[16] Bhat R.M., Hegde V., Budagumpi S., Adimule V., Keri R.S. (2024). Benzimidazole–Oxadiazole Hybrids—Development in Medicinal Chemistry: An Overview. Chem. Biol. Drug Des..

[17] Qiu J., Zou Y., Li S., Yang L., Qiu Z., Kong F., Gu X. (2022). Discovery of Benzimidazole Substituted 1, 2, 4-Oxadiazole Compounds as Novel Anti-HBV Agents with TLR8-Agonistic Activities. Eur. J. Med. Chem..

[18] Soni N., Soni N., Gupta P. (2016). Synthesis and In Vitro Anthelmintic Activity of Novel Substituted Oxadiazole Bearing Benzimidazole Derivatives. Der Pharma Chemica.

[19] Celik I., Sarıaltın S.Y., Çoban T., Kılcıgil G. (2022). Design, Synthesis, in Vitro and in Silico Studies of Benzimidazole-Linked Oxadiazole Derivatives as Anti-inflammatory Agents. ChemistrySelect.

[20] Hagar F.F., Abbas S.H., Sayed A.M., Gomaa H.A.M., Youssif B.G.M., Abdelhamid D., Abdel-Aziz M. (2025). New Antiproliferative 1,3,4-Oxadiazole/Benzimidazole Derivatives: Design, Synthesis, and Biological Evaluation as Dual EGFR and BRAFV600E Inhibitors. Bioorg. Chem..

[21] Çevik U.A., Celik I., Görgülü Ş., Şahin İnan Z.D., Bostancı H.E., Karayel A., Özkay Y., Kaplancıklı Z.A. (2025). Novel Benzimidazole–Oxadiazole Derivatives as Anticancer Agents with VEGFR2 Inhibitory Activity: Design, Synthesis, In Vitro Anticancer Evaluation, and In Silico Studies. ACS Omega.

[22] Çevik U.A., Osmaniye D., Çavuşoğlu B.K., Sağlik B.N., Levent S., Ilgin S., Can N.Ö., Özkay Y., Kaplancikli Z.A. (2019). Synthesis of Novel Benzimidazole–Oxadiazole Derivatives as Potent Anticancer Activity. Med. Chem. Res..

[23] Shruthi N., Poojary B., Kumar V., Hussain M.M., Rai V.M., Pai V.R., Bhat M., Revannasiddappa B.C. (2016). Novel Benzimidazole–Oxadiazole Hybrid Molecules as Promising Antimicrobial Agents. RSC Adv..

[24] Salahuddin, Shaharyar M., Mazumder A., Abdullah M.M. (2017). Synthesis, Characterization and Antimicrobial Activity of 1,3,4-Oxadiazole Bearing 1H-Benzimidazole Derivatives. Arab. J. Chem..

[25] Patel M., Avashthi G., Gacem A., Alqahtani M.S., Park H.-K., Jeon B.-H. (2023). A Review of Approaches to the Metallic and Non-Metallic Synthesis of Benzimidazole (BnZ) and Their Derivatives for Biological Efficacy. Molecules.

[26] Acar Çevik U., Celik I., Görgülü Ş., Şahin Inan Z.D., Bostancı H.E., Özkay Y., Kaplacıklı Z.A. (2024). New Benzimidazole-oxadiazole Derivatives as Potent VEGFR-2 Inhibitors: Synthesis, Anticancer Evaluation, and Docking Study. Drug Dev. Res..

[27] Tantray M.A., Khan I., Hamid H., Alam M.S., Dhulap A., Kalam A. (2016). Synthesis of Benzimidazole-Based 1,3,4-Oxadiazole-1,2,3-Triazole Conjugates as Glycogen Synthase Kinase-3β Inhibitors with Antidepressant Activity in in Vivo Models. RSC Adv..

[28] Acar Çevik U., Sağlık B.N., Osmaniye D., Levent S., Kaya Çavuşoğlu B., Karaduman A.B., Atlı Eklioğlu Ö., Özkay Y., Kaplancıklı Z.A. (2020). Synthesis, Anticancer Evaluation and Molecular Docking Studies of New Benzimidazole-1,3,4-Oxadiazole Derivatives as Human Topoisomerase Types I Poison. J. Enzym. Inhib. Med. Chem..

[29] Almalki A.S., Nazreen S., Elbehairi S.E.I., Asad M., Shati A.A., Alfaifi M.Y., Alhadhrami A., Elhenawy A.A., Alorabi A.Q., Asiri A.M. (2022). Design, Synthesis, Anticancer Activity and Molecular Docking Studies of New Benzimidazole Derivatives Bearing 1,3,4-Oxadiazole Moieties as Potential Thymidylate Synthase Inhibitors. New J. Chem..

[30] Bhasker G., Salahuddin, Mazumder A., Kumar R., Kumar G., Ahsan M.J., Shahar Yar M., Khan F., Kapoor B. (2024). Hybrids of Benzimidazole-Oxadiazole: A New Avenue for Synthesis, Pharmacological Activity and Recent Patents for the Development of More Effective Ligands. Curr. Org. Synth..

[31] Fathi M.A.A., Abd El-Hafeez A.A., Abdelhamid D., Abbas S.H., Montano M.M., Abdel-Aziz M. (2019). 1,3,4-Oxadiazole/Chalcone Hybrids: Design, Synthesis, and Inhibition of Leukemia Cell Growth and EGFR, Src, IL-6 and STAT3 Activities. Bioorg. Chem..

[32] Hagar F.F., Abbas S.H., Gomaa H.A.M., Youssif B.G.M., Sayed A.M., Abdelhamid D., Abdel-Aziz M. (2023). Chalcone/1,3,4-Oxadiazole/Benzimidazole Hybrids as Novel Anti-Proliferative Agents Inducing Apoptosis and Inhibiting EGFR & BRAFV600E. BMC Chem..

[33] Glomb T., Świątek P. (2021). Antimicrobial Activity of 1,3,4-Oxadiazole Derivatives. Int. J. Mol. Sci..

[34] Alzahrani H., Alam M., Elhenawy A., Nazreen S. (2022). Synthesis, Antimicrobial, Antiproliferative, and Docking Studies of 1,3,4-Oxadiazole Derivatives Containing Benzimidazole Scaffold. Biointerface Res. Appl. Chem..

[35] Akhter G., Hamid H., Dhawan B., Kumar Das A., Tantray M.A., Alam M.S., Sharma K. (2024). Synthesis and Design of New 1,3,4-Oxadiazole Benzimidazole Hybrids as Potential Antibacterial Agents Against MRSA by Targeting FabI. ChemistrySelect.

[36] Karaburun A.Ç., Kaya Çavuşoğlu B., Acar Çevik U., Osmaniye D., Sağlık B.N., Levent S., Özkay Y., Atlı Ö., Koparal A.S., Kaplancıklı Z.A. (2019). Synthesis and Antifungal Potential of Some Novel Benzimidazole-1,3,4-Oxadiazole Compounds. Molecules.

[37] Sousa C.F., Coimbra J.T.S., Ferreira M., Pereira-Leite C., Reis S., Ramos M.J., Fernandes P.A., Gameiro P. (2021). Passive Diffusion of Ciprofloxacin and Its Metalloantibiotic: A Computational and Experimental Study. J. Mol. Biol..

[38] Wang J., Ansari M.F., Zhou C.-H. (2021). Unique Para-Aminobenzenesulfonyl Oxadiazoles as Novel Structural Potential Membrane Active Antibacterial Agents towards Drug-Resistant Methicillin Resistant Staphylococcus Aureus. Bioorg. Med. Chem. Lett..

[39] Hu Z., Marti J. (2022). In Silico Drug Design of Benzothiadiazine Derivatives Interacting with Phospholipid Cell Membranes. Membranes.

[40] Aragón-Muriel A., Liscano Y., Morales-Morales D., Polo-Cerón D., Oñate-Garzón J. (2021). A Study of the Interaction of a New Benzimidazole Schiff Base with Synthetic and Simulated Membrane Models of Bacterial and Mammalian Membranes. Membranes.

[41] Hussein K.B. (2022). Synthesis, Antibacterial Activity of Sm(III) Complex with L-Phenylalanine. Zanco J. Pure Appl. Sci..

[42] Asadpour S., Aramesh-Boroujeni Z., Jahani S. (2020). In Vitro Anticancer Activity of Parent and Nano-Encapsulated Samarium(III) Complex towards Antimicrobial Activity Studies and FS-DNA/BSA Binding Affinity. RSC Adv..

[43] Cota I., Marturano V., Tylkowski B. (2019). Ln Complexes as Double Faced Agents: Study of Antibacterial and Antifungal Activity. Coord. Chem. Rev..

[44] Ain Q., Pandey S.K., Pandey O.P., Sengupta S.K. (2015). Synthesis, Spectroscopic, Thermal and Antimicrobial Studies of Neodymium(III) and Samarium(III) Complexes Derived from Tetradentate Ligands Containing N and S Donor Atoms. Spectrochim. Acta A Mol. Biomol. Spectrosc..

[45] Dzulkifli N.N., Farina Y., Yamin B.M., Ibrahim N. (2017). Synthesis, structural, chemical properties, and anti-bacterial screening of sm(iii) thiosemicarbazone complexes. Malays. J. Anal. Sci..

[46] Moreno-Ramirez M.C., Arias-Bravo A.S., Aragón-Muriel A., Godoy C.A., Liscano Y., Garzón J.O., Polo-Cerón D. (2024). Design, Synthesis and Antimicrobial Potential of Conjugated Metallopeptides Targeting DNA. Sci. Pharm..

[47] Aragón-Muriel A., Aguilar-Castillo B.A., Rufino-Felipe E., Valdés H., González-Sebastián L., Osorio-Yáñez R.N., Liscano Y., Gómez-Benítez V., Polo-Cerón D., Morales-Morales D. (2022). Antibacterial Activity and Molecular Studies of Non-Symmetric POCOP-Pd(II) Pincer Complexes Derived from 2,4-Dihydroxybenzaldehyde (2,4-DHBA). Polyhedron.

[48] Aragón-Muriel A., Liscano Y., Upegui Y., Robledo S.M., Ramírez-Apan M.T., Morales-Morales D., Oñate-Garzón J., Polo-Cerón D. (2021). In Vitro Evaluation of the Potential Pharmacological Activity and Molecular Targets of New Benzimidazole-Based Schiff Base Metal Complexes. Antibiotics.

[49] Aragón-Muriel A., Liscano-Martínez Y., Rufino-Felipe E., Morales-Morales D., Oñate-Garzón J., Polo-Cerón D. (2020). Synthesis, Biological Evaluation and Model Membrane Studies on Metal Complexes Containing Aromatic N,O-Chelate Ligands. Heliyon.

[50] Husain A., Rashid M., Mishra R., Parveen S., Shin D.-S., Kumar D. (2012). Benzimidazole Bearing Oxadiazole and Triazolo-Thiadiazoles Nucleus: Design and Synthesis as Anticancer Agents. Bioorg. Med. Chem. Lett..

[51] Váquiro-Reyes I.Y., Aragón-Muriel A., Polo-Cerón D. (2019). Síntesis, Caracterización y Evaluación Farmacológica de Nuevos Complejos Metálicos Derivados de Híbridos Heteroaromáticos (Benzimidazol/Oxadiazol). Rev. Colomb. Cienc. Químico-Farm..

[52] Koeth L.M., Miller L.A. (2023). Antimicrobial Susceptibility Test Methods: Dilution and Disk Diffusion Methods. ClinMicroNow.

[53] Boon J.M., Smith B.D. (2002). Chemical Control of Phospholipid Distribution across Bilayer Membranes. Med. Res. Rev..

[54] Escribá P.V., González-Ros J.M., Goñi F.M., Kinnunen P.K.J., Vigh L., Sánchez-Magraner L., Fernández A.M., Busquets X., Horváth I., Barceló-Coblijn G. (2008). Membranes: A Meeting Point for Lipids, Proteins and Therapies. J. Cell Mol. Med..

[55] Madigan M., Sattley W., Aiyer J., Stahl D., Buckley D. (2021). Brock Biology of Microorganisms, Global Edition.

[56] Böttcher C.J.F., Van Gent C.M., Pries C. (1961). A Rapid and Sensitive Sub-Micro Phosphorus Determination. Anal. Chim. Acta.

[57] Ausili A., Gómez-Murcia V., Candel A.M., Beltrán A., Torrecillas A., He L., Jiang Y., Zhang S., Teruel J.A., Gómez-Fernández J.C. (2021). A Comparison of the Location in Membranes of Curcumin and Curcumin-Derived Bivalent Compounds with Potential Neuroprotective Capacity for Alzheimer’s Disease. Colloids Surf. B Biointerfaces.

[58] Pabst G., Rappolt M., Amenitsch H., Laggner P. (2000). Structural Information from Multilamellar Liposomes at Full Hydration: Full q-Range Fitting with High Quality x-Ray Data. Phys. Rev. E.

[59] Ausili A., Martínez-Valera P., Torrecillas A., Gómez-Murcia V., de Godos A.M., Corbalán-García S., Teruel J.A., Gómez Fernández J.C. (2018). Anticancer Agent Edelfosine Exhibits a High Affinity for Cholesterol and Disorganizes Liquid-Ordered Membrane Structures. Langmuir.

[60] Ramos R.S., Borges R.S., de Souza J.S.N., Araujo I.F., Chaves M.H., Santos C.B.R. (2022). Identification of Potential Antiviral Inhibitors from Hydroxychloroquine and 1,2,4,5-Tetraoxanes Analogues and Investigation of the Mechanism of Action in SARS-CoV-2. Int. J. Mol. Sci..

[61] Hay R.W. (2000). The Kinetic Background. Reaction Mechanisms of Metal Complexes.

[62] Maciuca A.-M., Munteanu A.-C., Mihaila M., Badea M., Olar R., Nitulescu G.M., Munteanu C.V.A., Bostan M., Uivarosi V. (2020). Rare-Earth Metal Complexes of the Antibacterial Drug Oxolinic Acid: Synthesis, Characterization, DNA/Protein Binding and Cytotoxicity Studies. Molecules.

[63] Bistoni G., Rampino S., Scafuri N., Ciancaleoni G., Zuccaccia D., Belpassi L., Tarantelli F. (2016). How π Back-Donation Quantitatively Controls the CO Stretching Response in Classical and Non-Classical Metal Carbonyl Complexes. Chem. Sci..

[64] Patel M.N., Pansuriya P.B., Parmar P.A., Gandhi D.S. (2008). Synthesis, Characterization, and Thermal and Biocidal Aspects of Drug-Based Metal Complexes. Pharm. Chem. J..

[65] Kim W.K., An J.M., Lim Y.J., Kim K., Kim Y.H., Kim D. (2025). Recent Advances in Metallodrug: Coordination-Induced Synergy between Clinically Approved Drugs and Metal Ions. Mater. Today Adv..

[66] Casal H.L., Mantsch H.H. (1984). Polymorphic Phase Behaviour of Phospholipid Membranes Studied by Infrared Spectroscopy. Biochim. Biophys. Acta (BBA)-Rev. Biomembr..

[67] Lewis R.N.A.H., Prenner E.J., Kondejewski L.H., Flach C.R., Mendelsohn R., Hodges R.S., McElhaney R.N. (1999). Fourier Transform Infrared Spectroscopic Studies of the Interaction of the Antimicrobial Peptide Gramicidin S with Lipid Micelles and with Lipid Monolayer and Bilayer Membranes. Biochemistry.

[68] Tardieu A., Luzzati V., Reman F.C. (1973). Structure and Polymorphism of the Hydrocarbon Chains of Lipids: A Study of Lecithin-Water Phases. J. Mol. Biol..

[69] Holland J.W., Cullis P.R., Madden T.D. (1996). Poly(Ethylene Glycol)–Lipid Conjugates Promote Bilayer Formation in Mixtures of Non-Bilayer-Forming Lipids. Biochemistry.

[70] Sharma B., Shukla S., Rattan R., Fatima M., Goel M., Bhat M., Dutta S., Ranjan R.K., Sharma M. (2022). Antimicrobial Agents Based on Metal Complexes: Present Situation and Future Prospects. Int. J. Biomater..

[71] Lewis R.N.A.H., Mannock D.A., McElhaney R.N. (2007). Differential Scanning Calorimetry in the Study of Lipid Phase Transitions in Model and Biological Membranes. Methods in Membrane Lipids.

[72] Leekumjorn S., Sum A.K. (2007). Molecular Studies of the Gel to Liquid-Crystalline Phase Transition for Fully Hydrated DPPC and DPPE Bilayers. Biochim. Biophys. Acta (BBA)-Biomembr..

[73] Neunert G., Tomaszewska-Gras J., Baj A., Gauza-Włodarczyk M., Witkowski S., Polewski K. (2021). Phase Transitions and Structural Changes in DPPC Liposomes Induced by a 1-Carba-Alpha-Tocopherol Analogue. Molecules.

[74] Lewis R.N., McElhaney R.N., Pohle W., Mantsch H.H. (1994). Components of the Carbonyl Stretching Band in the Infrared Spectra of Hydrated 1,2-Diacylglycerolipid Bilayers: A Reevaluation. Biophys. J..

[75] Rappolt M., Hickel A., Bringezu F., Lohner K. (2003). Mechanism of the Lamellar/Inverse Hexagonal Phase Transition Examined by High Resolution X-Ray Diffraction. Biophys. J..

[76] Rand R.P., Luzzati V. (1968). X-Ray Diffraction Study in Water of Lipids Extracted from Human Erythrocytes. Biophys. J..

[77] Roessle M., Svergun D.I. (2013). Small Angle X-Ray Scattering. Encyclopedia of Biophysics.

[78] Hauser H. (1984). Some Aspects of the Phase Behaviour of Charged Lipids. Biochim. Biophys. Acta (BBA)-Biomembr..

[79] Pabst G., Danner S., Karmakar S., Deutsch G., Raghunathan V.A. (2007). On the Propensity of Phosphatidylglycerols to Form Interdigitated Phases. Biophys. J..

[80] Hamley I.W. (2022). Diffuse Scattering from Lamellar Structures. Soft Matter.

[81] Valtersson C., van Duÿn G., Verkleij A.J., Chojnacki T., de Kruijff B., Dallner G. (1985). The Influence of Dolichol, Dolichol Esters, and Dolichyl Phosphate on Phospholipid Polymorphism and Fluidity in Model Membranes. J. Biol. Chem..

[82] Luzzati V., Gulik-Krzywicki T., Rivas E., Reiss-Husson F., Rand R.P. (1968). X-Ray Study of Model Systems: Structure of the Lipid-Water Phases in Correlation with the Chemical Composition of the Lipids. J. Gen. Physiol..

[83] Oliveira L.P.S., Lima L.R., Silva L.B., Cruz J.N., Ramos R.S., Lima L.S., Cardoso F.M.N., Silva A.V., Rodrigues D.P., Rodrigues G.S. (2023). Hierarchical Virtual Screening of Potential New Antibiotics from Polyoxygenated Dibenzofurans against Staphylococcus Aureus Strains. Pharmaceuticals.

[84] Halimi Syla G., Osmaniye D., Korkut Çelikateş B., Özkay Y., Kaplancıklı Z.A. (2025). Targeting Cancer with New Morpholine-Benzimidazole-Oxadiazole Derivatives: Synthesis, Biological Evaluation, and Computational Insights. ACS Omega.

[85] Ding X., Fan L., Wang L., Zhou M., Wang Y., Zhao Y. (2023). Designing Self-Healing Hydrogels for Biomedical Applications. Mater. Horiz..

[86] Mandour H.S.A., Rehab A., Elnahrawy M., Salahuddin N. (2025). Synthesis, Characterization and Bioactivity of Chalcone-Based Polybenzoxazine/Copper Oxide Nanocomposites. RSC Adv..

[87] Fijałkowski P., Impert O., Pomastowski P., Rafińska K., Walczak-Skierska J., Fijałkowski P., Katafias A., van Eldik R. (2025). Synthesis of Nanoparticles of Cobalt Protoporphyrin IX (Co(iii) PPIX NPs). Antiradical, Cytotoxicity and Antibacterial Properties. RSC Adv..

[88] Movahedi F., Gu W., Soares C.P., Xu Z.P. (2021). Encapsulating Anti-Parasite Benzimidazole Drugs into Lipid-Coated Calcium Phosphate Nanoparticles to Efficiently Induce Skin Cancer Cell Apoptosis. Front. Nanotechnol..

