# Supplementary material for: Heteroaromatic Hybrid Benzimidazole/Oxadiazole (BZ/OZ) Ligand and Its Sm(III) Complex: Study of Their Antibacterial Activity, Toxicological Prediction and Interaction with Different Model Membranes
Source: Biomolecules. 2025 Nov 7;15(11):1568. doi: 10.3390/biom15111568 (PMC12650029; doi:10.3390/biom15111568)
Supplement: Supplementary file 1 [file biomolecules-15-01568-s001.zip › biomolecules-3937444-supplementary.pdf]

Supporting Information for:

Heteroaromatic Hybrid Benzimidazole/Oxadiazole (BZ/OZ) Ligand and Its Sm(III) Complex: Study of Their Antibacterial Activity, Toxicological Prediction and Interaction with Different Model Membranes

Alberto Aragón-Muriel [1], Alessio Ausili [2,*], Luciana Sampaio Lima [3], Cleydson B. R. Santos [3], David Morales-Morales [4] and Dorian Polo-Cerón [5,*]

[1] Grupo de Investigaciones Bioquímicas (GIB), Departamento de Química, Universidad del Magdalena, Santa Marta 470004, Colombia; aaragonm@unimagdalena.edu.co

[2] Departamento de Bioquímica y Biología Molecular (A), Facultad de Veterinaria, International Campus of Excellence Mare Nostrum, Universidad de Murcia, Apartado. 4021, E-30100 Murcia, Spain

[3] Laboratório de Modelagem e Química Computacional, Universidade Federal do Amapá, Macapá 68903-419, Amapá, Brazil; lucianasampaio@unifap.br (L.S.L.); breno@unifap.br (C.B.R.S.)

[4] Instituto de Química, Universidad Nacional Autónoma de México, Circuito Exterior S/N, Ciudad Universitaria, Alcaldía Coyoacán, Ciudad de México C.P. 04510, Mexico; damor@unam.mx

[5] Laboratorio de Investigación en Catálisis y Procesos (LICAP-Bioinorgánica), Departamento de Química, Facultad de Ciencias Naturales y Exactas, Universidad del Valle, Calle 13 No. 100-00, Santiago de Cali 76001, Colombia

* Correspondence: aausili@um.es (A.A.); dorian.polo@correounivalle.edu.co (D.P.-C.)

List of Figures;

Figure S1: FT-IR spectra of precursor hydrazide, ligand (L), and samarium complex.
Figure S2: ^{1}H NMR spectra of benzimidazole/oxadiazole ligand (L).

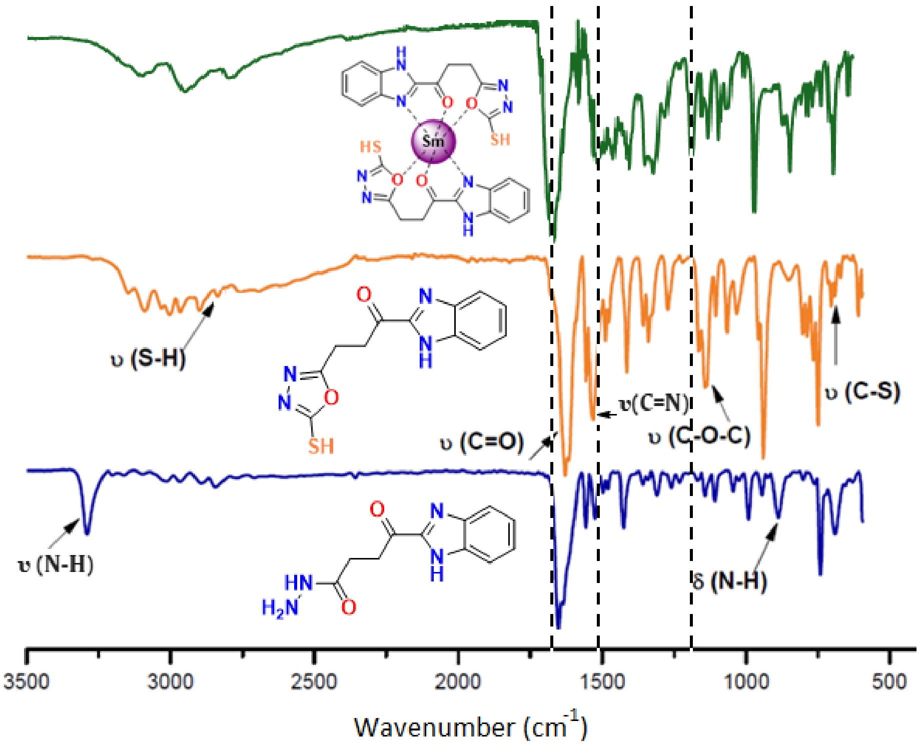

Figure S1. FT-IR spectra of precursor hydrazide, ligand (L), and samarium complex.

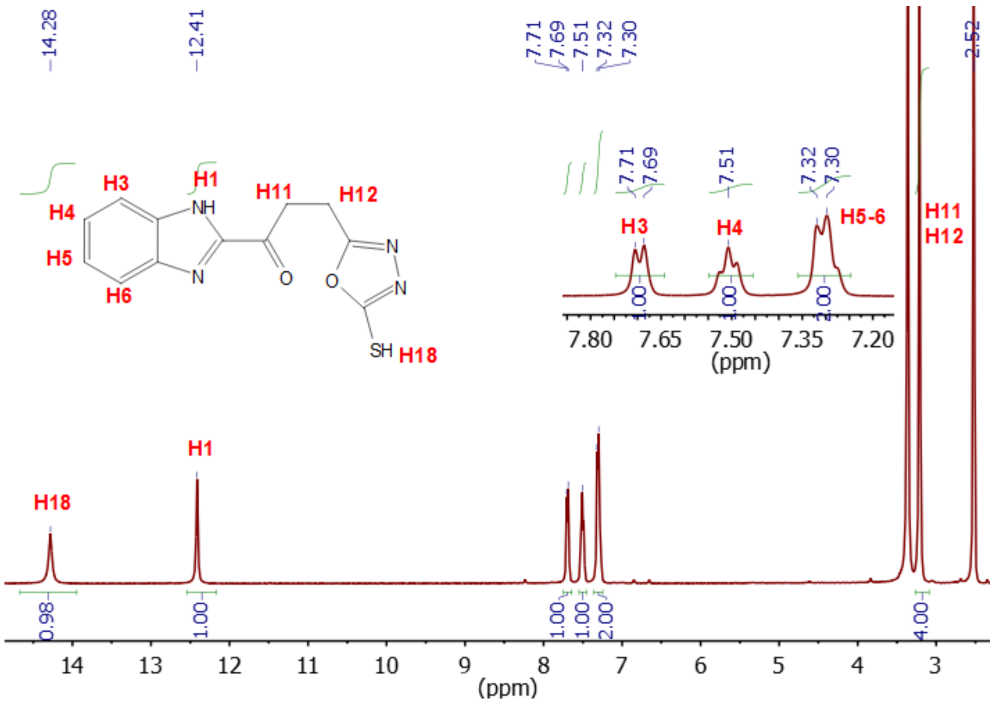

Figure S2. ^{1}H NMR spectra of benzimidazole/oxadiazole ligand (L).
